# Genomic, metabolomic, and functional properties of probiotic lactic acid bacteria isolated from Indonesian stingless bee honey

**DOI:** 10.1007/s10123-026-00794-4

**Published:** 2026-03-13

**Authors:** Ema Damayanti, Tsania Taskia Nabila, Nur Fitrianto, Yeyen Prestyaning Wanita, Fenny Amilia Mahara, Vika Tresnadiana Herlina, Vita Taufika Rosyida, Teguh Wahyono, Muslih Anwar, Rofiq Sunaryanto, Puspita Lisdiyanti

**Affiliations:** 1 Research Center for Food Technology and Processing, National Research and Innovation Agency (BRIN), Jl. Jogja – Wonosari KM 31.5, Gunungkidul, Yogyakarta Indonesia; 2 Indonesian Biofilm Research Collaboration Centre (IBRCC), Faculty of Medicine, Public Health, and Nursing, Universitas Gadjah Mada, Sleman, Yogyakarta Indonesia; 3 Doctoral Student of Pharmacy, Faculty of Pharmacy, Universitas Gadjah Mada, Sleman, Yogyakarta Indonesia; 4 Research Assistant of Probiotic and Food Bioprocess Research Group, Research Center for Food Technology and Processing, National Research and Innovation Agency (BRIN), Gunungkidul, Yogyakarta Indonesia; 5 Research Center for Biosystematic and Evolution, National Research and Innovation Agency (BRIN), Cibinong, West Java Indonesia

**Keywords:** Antioxidant, Lactic acid bacteria, Metabolomic, Probiotic, Stingless bee honey

## Abstract

Stingless bee honey (SBH) is recognized as a functional food with antibacterial, antioxidant, and anticancer properties. This study aimed to isolate and characterize lactic acid bacteria (LAB) with potential probiotic, antibacterial, and antioxidant activities from SBH. LAB were isolated from honey samples of seven Indonesian stingless bee species and screened for antibacterial activity. Five isolates with the highest antibacterial activity were selected for further characterization, including whole genome sequencing (WGS), probiotic property assessment, antioxidant activity, α-amylase inhibitory potential of their cell-free supernatants (CFS), and metabolomic profiling. The isolates were identified as *Lacticaseibacillus rhamnosus* (TB-3, HI-1, and TS-4) and *Pediococcus acidilactici* (HI-5 and LT-3) based on WGS analysis. Genome-mining revealed biosynthetic gene clusters (BGCs), including T3PKS, RiPP-like and bacteriocin. All isolates exhibited good probiotic properties with high survival rates under simulated gastrointestinal conditions. CFS of the isolates exhibited strong antibacterial activity against *Escherichia coli*, *Staphylococcus aureus*, and *Pseudomonas aeruginosa*, as well as significant antioxidant capacity (DPPH, ABTS, and FRAP), high polyphenol and flavonoid contents, and α-amylase inhibition. Untargeted metabolomic analysis using UHPLC-HRMS revealed distinct metabolite profiles with several potential bioactive compounds. This study highlights the potential of SBH-derived LAB as probiotic candidates with antibacterial and antioxidant properties. The integration of genomic and metabolomic approaches offers valuable insights into their application in functional foods and nutraceuticals.

**Supplementary Information:**

The online version contains supplementary material available at 10.1007/s10123-026-00794-4.

## Introduction

The most widely recognized honey-producing bees are honey bees and stingless bees (SBs). While honey bees belong to the genus *Apis*, SBs are primarily classified within the genera *Melipona* and *Trigona* (Zulkhairi Amin et al. 2018, 2019). In contrast to *Apis* bees, SBs are characterized by the absence of a functional sting, a trait reflected in their common name. They typically fly short distances, and predominantly to collect nectar from creeping flowers (Braghini et al. 2022) such as coral vine, *Antigonon leptopus* (Damayanti et al. 2025), *Asystasia gangentica micrantha*, and *Ipomea nil* (Basari et al., 2021) which contributes to the distinctive characteristics of their honey (Vit et al. 2025). Based on current taxonomic records, there are 605 described extant species of SBs distributed across 45 genera worldwide (Engel et al. 2023), and nearly 600 of these species are known to produce tropical pot-honey, processed and stored in cerumen pots (Vit et al. 2023).

Stingless bee honey (SBH), also referred to as pot-honey, is a distinct natural product that differs from *Apis* honey in terms of flavor profile, chemical composition, biological properties, and acidity (Gadge et al. 2023). Compared to *Apis mellifera* honey, SBH typically exhibits higher moisture content, higher acidity, and lower sugar concentration (Santos et al. 2021). These differences are attributed to its unique physicochemical composition, functional attributes, and the specific biological traits of SBs. The quality and characteristics of SBH are influenced by multiple factors, including floral sources, geographical origin, climate, bee species, and post-harvest handling and storage conditions (Gadge et al. 2023). Although sugars constitute the main component, SBH also contains high moisture content, organic acids, proteins, amino acids, enzymes, vitamins, minerals, and bioactive compounds such as phenolics and flavonoids (Biluca et al. 2016). Beyond its nutritional value, SBH is also recognized for its potential medicinal applications (Zulkhairi Amin et al. 2018; Rosli et al. 2020; Braghini et al., 2022; Gadge et al., 2024; Vit et al., 2025).

The unique composition of SBH (17.3–42.7% of moisture, 4.95–101 mEq/kg of free acidity and 25.0–67.8 g/100 g of reducing sugar), particularly its content of various bioactive compounds, such as high of ascorbic acid, phenolic acids flavonoids, tocopherol and enzymes, contributes to its diverse health-promoting effects (Alves et al. 2024). Honey, in general, remains underexplored as a natural source of bioactive compounds with potential applications in food, medicine, and human health (Alves et al. 2024). SBH has been reported to exhibit various nutraceutical properties, including anticataract, anti-inflammatory, antibacterial, antioxidant, antiproliferative, and neuroprotective activities, supporting its use as a functional food supplement (Vit et al. 2025). The physicochemical, functional, and microbiological properties of SBH are strongly influenced by the SB species involved (Melia et al. 2024). For instance, honey derived from *Heterotrigona itama* and *Tetragonula laeviceps* has demonstrated antibacterial, antifungal, antioxidant, and anticancer activities (Damayanti et al. 2024) while Malaysian *Trigona* honey has shown potent antibacterial and bactericidal effects against pathogenic bacteria (Al-Kafaween et al. 2020). Bioactive components in SBH may originate from environmental sources, the bees themselves, or the metabolic activity of associated symbiotic microorganisms (Alves et al. 2024). Numerous studies have demonstrated that these microbial communities may not only contribute to bee health but also enhance the bioactivity of SBH (Tsadila et al. 2023). Moreover, the nutraceutical potential of bioactive compounds in SBH has been reported to vary depending on the composition of its associated microbes (Vit et al. 2025). Therefore, exploring the microbial communities present in SBH, particularly those with probiotic potential, is important to better understand their contribution to the honey’s functional properties and their prospective applications in health-promoting formulations.

SBH is increasingly recognized as a complex microbial niche, particularly rich in lactic acid bacteria (LAB), with dominant genera associated with bees such as *Apilactobacillus*, *Bombilactobacillus*, *Lactobacillus*, and *Fructobacillus* (Oliphant et al. 2022). Microbial profiling of SBH from eight SB species revealed a total of 70 bacterial species across 155 genera, with *Lactobacillus malefermentans* emerging as a dominant and potentially native bacterium consistently found across all samples (Rosli et al. 2020). These findings highlight the potential of SBH to harbor LAB strains with distinct metabolic adaptations and functional capabilities. Recent studies have identified fructophilic LAB (FLAB), such as *Fructobacillus pseudoficulneus*, *F. tropaeoli*, and *Fructilactobacillus* spp., from four SB species (Andrade-Velásquez et al. 2023), as well as novel LAB species associated with Australian SBs, namely *Apilactobacillus apisilvae*, *Bombilactobacillus thymidiniphilus*, *B. folatiphilus*, and *Nicolia spurrieriana* (Oliphant et al. 2022).

The presence of LAB in SBH may contribute not only to its preservation but also to its functional properties through the production of bioactive metabolites. For example, Melia et al. (2024) reported LAB with antimicrobial and antioxidant activity in honey derived from indigenous West Sumatran SB species, such as *Heterotrigona itama*, *Geniotrigona thoracica*, *Tetragonula melanoleuca*, and *T. binghami*. Despite this potential, studies on LAB diversity and function in Indonesian SBH remain limited. To address this gap, the present study explores the genomic and metabolomic profiles of LAB from seven indigenous stingless bee species, namely *Heterotrigona itama*, *Tetragonula laeviceps*, *T. clypearis*, *T. sarawakensis*, *Lepidotrigona terminata*, *T. drescheri*, and *T. biroi*. Further investigations into their metabolic profiles and genetic features may uncover novel mechanisms and probiotic strains with enhanced health benefits. Moreover, studying the molecular basis of their antimicrobial and antioxidant activities could facilitate targeted strain selection and genetic optimization, thereby advancing their application in food biotechnology and preservation. This study was explored genome-resolved and metabolomics-integrated analysis of LAB from Indonesian SBH. This study demonstrated the first report of α-amylase inhibitory activity of SBH-derived LAB. Discovery of specific biosynthetic gene clusters (T3PKS, RiPP, NRPS) linked to functional activities also identified in this study.

## Materials and methods

### Sample collection and honey preparation

Honey samples were collected from beekeepers in the Bantul Region, Yogyakarta Province, Indonesia. The samples were derived from seven SB species, including *Heterotrigona itama*, *Tetragonula laeviceps*, *T. clypearis*, *T. sarawakensis*, *Lepidotrigona terminata*, *T. dresceri*, and *T. biroi*. Honey from *H. itama* was harvested using a suction machine, whereas honey from the other species was manually extracted from honey pots by squeezing under sterile conditions, as described by (Damayanti et al. 2024). All honey samples were transferred into sterile bottles, harvested in August 2023, and stored at room temperature until further analysis.

### Isolation and initial characterization of lactic acid bacteria (LAB)

Five grams of honey from each stingless bee honey sample were diluted in 45 mL of sterile 0.86% NaCl solution (Merck, Germany), homogenized, and serially diluted up to 10^4^. Bacterial isolates were obtained by streaking onto De Man, Rogosa, and Sharpe Agar (MRSA; Merck, Germany) and incubated at 37 °C for 3 days under microaerophilic conditions to support LAB growth. Colonies were then purified to obtain single-colony isolates and subjected to morphological observation, Gram staining, catalase testing, and molecular identification. Macroscopic morphology was observed using a light microscope, while microscopic features were examined using a scanning electron microscope (SEM; Hitachi, Japan). Isolates were preserved in glycerol stock and stored at -80 °C in a deep freezer (Thermo Scientific) prior to analysis.

### Screening for antibacterial activity

Screening for antibacterial activity was conducted to identify LAB isolates with the highest antibacterial activity. This selection guided subsequent analyses (genome sequencing, probiotic characterization, and metabolomic profiling) by focusing on the most bioactive candidates with potential relevance in antimicrobial and probiotic applications. The assay was performed using cell-free supernatant (CFS), prepared by culturing LAB isolates (10^7^ CFU/mL) in MRS broth at 37 °C for 24 h. Cultures were centrifuged (Megafuge™, Thermo Scientific™, USA) at 4 °C for 10 min at 4137 × g, and filtered through a 0.45 μm sterile syringe filter (Corning, Germany) to obtain the CFS. The freshly prepared CFS was used immediately for antibacterial assays.

Antibacterial activity of the CFS was assessed using the microdilution method (Damayanti et al. 2014) against three pathogenic indicator strains: *Escherichia coli* FNCC 0091, *Pseudomonas aeruginosa* FNCC 0070, and *Staphylococcus aureus* FNCC 0047. All pathogenic bacterial strains were cultured overnight at 37 °C in Brain Heart Infusion (BHI) broth (Himedia, India), and the bacterial inoculum was standardized to 10⁷ CFU/mL.

The assay was conducted in sterile 96-well microplates (Iwaki, Japan), with each well receiving 100 µL of CFS (prepared at 30% v/v in BHI) and 5% (v/v) pathogenic bacterial inoculum. BHI medium without CFS served as a negative control. Sterile BHI medium without bacteria was used as the blank. All treatments were conducted in triplicate. Plates were incubated aerobically at 37 °C for 24 h. Absorbance was measured at 600 nm using a microplate reader (Multiskan GO™, Thermo Scientific, USA) at both 0 and 24 h. The percentage of bacterial growth inhibition was calculated using the following formula:

$$inhibition(\%)=\lbrack(A-B)/A\rbrack\cdot100,$$

where *A* is the absorbance of the negative control (without CFS), and *B* is the absorbance of the treated sample (with CFS).

### Molecular identification of LAB isolates

Molecular identification of LAB isolates was performed through 16S rRNA gene analysis. Genomic DNA (gDNA) was extracted using the FavorPrep DNA Isolation Mini Kit (Favorgen Biotech Corp, Taiwan). PCR amplification of the 16S rRNA gene was conducted using universal primers 27F (5’-AGAGTTTGATCCTGGCTCAG-3’) and 1492R (5’-GGTTACCTTGTTACGACTT-3’) in a PCR thermal cycler, as previously described (Damayanti et al. 2012). PCR amplicons were visualized using agarose gel electrophoresis, and purified products were subjected to sequencing using the Sanger dideoxy method. The resulting sequences were compared against the NCBI database using BLAST to determine species-level homology.

### Genomic analysis of selected Lab isolates

#### Whole-genome sequencing, assembly, and annotation

The selected LAB isolates with the highest antibacterial activity were subjected to whole-genome sequencing. Library preparation of gDNA was carried out using the Oxford Nanopore Technologies (ONT) Library Preparation Kit (SQK-NBD114-24). The gDNA was end-repaired using an end-prep enzyme mix to generate 5’ phosphorylated and 3’ dA-tailed ends, followed by ligation with ONT-compatible adapters. The prepared library was quantified using a Qubit Fluorometer and loaded onto a FLO-MIN114 flow cell for sequencing on the PromethION platform (Oxford Nanopore Technologies). Sequencing was controlled by MinKNOW software (v23.11.7), and basecalling was performed in high-accuracy mode (HAC) using Dorado (v7.2.13). Raw reads were filtered using Filtlong (v0.2.1) based on length and quality, and their quality was assessed with NanoPlot (v1.40.2).

Read correction was performed using Canu (v2.2), followed by genome assembly using Flye (v2.8.3-b1695). The assembled genome was polished through four rounds of Racon (v1.5.0) and three rounds of Medaka (v1.7.2). Read mapping was conducted using Minimap2 (v2.24-r1122) to support the polishing steps. The quality of the assembled genome was evaluated using QUAST (v5.0.2) and Qualimap (v2.2.2-dev) to assess assembly metrics such as genome size, N50, and coverage statistics.

Genome annotation was performed using Prokka (Seeman, 2014), and a graphical circular genome map was generated using the Proksee server (https://proksee.ca/) (Grant et al. 2023). Additional functional annotation was conducted using the Rapid Annotations using Subsystems Technology (RAST) server (https://rast.nmpdr.org/; accessed on February 10, 2025) (Aziz et al. 2008; Overbeek et al. 2014). Subsystem classification and visualization of annotated gene functions were carried out using the SEED Viewer platform (https://pubseed.theseed.org/seedviewer.cgi; accessed on February 10, 2025) (Overbeek et al. 2014), which enabled the organization of coding sequences into biological functional categories.

#### Phylogenomic analysis

To perform whole-genome-based taxonomic analysis, including digital DNA–DNA hybridization (dDDH), genomic G + C content, and phylogenetic inference based on whole-genome sequencing, the assembled genome was analyzed using the Type (Strain) Genome Server (TYGS) (https://tygs.dsmz.de; accessed on March 25, 2025) (Meier-Kolthoff and Göker 2019). The resulting intergenomic distances were used to construct a balanced minimum evolution tree with branch support, inferred using FASTME 2.1.6.1 with subtree pruning and regrafting (SPR) post-processing (Lefort et al. 2015). Branch support values was calculated from 100 pseudo-bootstrap replicates. The tree was midpoint-rooted (Farris, 1972) and visualized using PhyD3 (Kreft et al. 2017).

Average nucleotide identity (ANI) was determined using the ANI calculator available at https://www.ezbiocloud.net/tools/ani (accessed on March 26, 2025). Genome-to-genome distance, ANI, and tetranucleotide signature correlations were computed across database genomes, and pairwise genomic comparisons were performed. Tetranucleotide frequencies (TETRA) and ANIb values were calculated using the JSpecies software package (v4.0.2) (https://jspecies.ribohost.com/jspeciesws/#analyse; accessed on March 25, 2025) (Richter et al. 2016). Species delineation thresholds were defined as ≥ 95% for ANIb and > 0.99 for TETRA values (Richter and Rosselló-Móra 2009).

#### Genome mining for bioactive compounds prediction

Genome mining of the LAB whole-genome sequence (WGS) was performed to identify biosynthetic gene clusters (BGCs) and putative secondary metabolites. AntiSMASH version 7.0.0 (https://antismash.secondarymetabolites.org/#!/start; accessed on March 26, 2025) was used to detect BGCs and predict associated metabolites (Blin et al. 2023). In addition, BAGEL4 (http://bagel4.molgenrug.nl/; accessed on March 26, 2025) was used to identify gene clusters potentially encoding bacteriocin or other ribosomally synthesized and post-translationally modified peptides (RiPPs) (Van Heel et al. 2018).

### Probiotic characterization of selected LAB isolates

A previously established method was employed to evaluate the probiotic properties of the selected LAB isolates with the highest antibacterial activity, including their tolerance to bile salts, simulated gastric juice, and low pH (Damayanti et al. 2012; Damayanti et al. 2025). One mL of 18-hour LAB culture (approximately 10⁸ CFU/mL or OD₆₀₀ ≈ 1.0) was centrifuged at 4 °C for 10 min at 4137 × g. The pellet was washed twice with sterile phosphate-buffered saline (PBS; Merck, Germany) and resuspended in 1 mL of PBS for subsequent assays.

For the bile salt tolerance assay, 0.2 mL of LAB suspension in PBS was mixed with 0.8 mL of PBS containing 0.3% (w/v) bile salts (Merck, Germany), resulting in a final volume of 1 mL. The mixture was incubated at 37 °C, and samples were collected at 0, 1, and 3 h. Viable cell counts were determined by serial dilution in PBS and plating on MRS Agar.

For the low-pH tolerance experiment, the washed LAB cells were resuspended in PBS and inoculated (0.2 mL) into 1.8 mL of MRS Broth that had been adjusted to pH 2 using 1 M HCl (Merck, Germany) (final volume: 2.0 mL; inoculum-to-broth ratio: 1:9). The mixture was incubated at 37 °C, and samples were collected at 0, 1, and 2 h for viable cell count analysis.

To assess tolerance to simulated gastric juice, the washed LAB cells were resuspended in 0.3 mL of PBS, and 0.2 mL of this suspension was added to 1 mL of artificial gastric juice prepared by dissolving 3 g/L of pepsin (Sigma Aldrich) in 0.85% NaCl and adjusting the pH to 2.0 using 1 M HCl, resulting in a final volume of 1.2 mL. After homogenization, the mixture was incubated at 37 °C, and samples were collected at 0, 1, and 3 h.

In all assays, samples were serially diluted and plated on MRS Agar for viable cell count determination. Plates were incubated at 37 °C for 48 h under microaerophilic conditions. Cell viability was calculated using the following formula:

$$cell\;viability(\%)=(B/A)\cdot100,$$

where *A* is the initial viable cell count (log_10_ CFU/mL) at 0 h, and *B* is the viable cell count after the incubation period. All treatments were performed in triplicate.

### Antioxidant activity assays of LAB cell-free supernatants (CFS)

#### 2,2-diphenyl-1-picrylhydrazyl (DPPH) Assay

The antioxidant activity of the LAB CFS was assessed using the DPPH (2,2-diphenyl-1-picrylhydrazyl) radical scavenging assay with slight modifications from a previously reported method (Wahyono et al. 2024). The CFS was initially diluted to 50% (v/v) in methanol (Merck, Germany), vortexed thoroughly, and centrifuged for 5 min at 4500 rpm. The resulting supernatant was filtered through a 0.22 μm PTFE syringe filter to obtain the test sample. To perform the assay, 80 µL of CFS at various concentrations (62.5%, 75%, 87.5%, and 100% v/v) were mixed with 20 µL of freshly prepared DPPH solution (0.4 mg/mL or approximately 100 µM; Himedia, India) in methanol (Merck, Germany). The mixtures were incubated in the dark at room temperature for 45 min to allow the reaction to occur. Methanol (80 µL) mixed with 20 µL of DPPH solution served as the negative control, while ascorbic acid (Merck, Germany) 2–32 µg/mL in methanol; was used as a standard. Each treatment was conducted in triplicates. After incubation, the absorbance was measured at 517 nm using a microplate reader (Multiskan GO^™^, Thermo Scientific, USA). The percentage of DPPH radical scavenging activity was calculated using the following formula:

$$scavenging\;activity\left(\%\right)=\left[\right(Ac-As)/Ac]\times100,$$

where *As* is the absorbance of the sample (CFS or standard antioxidant), and *Ac* is the absorbance of the negative control.

#### 2,2′-Azino-Bis-(3-Ethylbenzothiazoline-6-Sulfonic Acid (ABTS) Assay

The ABTS radical-scavenging activity of the LAB CFS samples was evaluated using a modified method based on a previously described protocol (Moussaoui et al. 2021). CFS samples were diluted with methanol (Merck, Germany) as described for the DPPH assay. To prepare the ABTS radical cation (ABTS•+), a stock solution containing 7 mM ABTS (Sigma Aldrich, USA) and 2.45 mM potassium persulfate (Merck, Germany) was mixed and incubated in the dark at room temperature for 16 h. After incubation, the ABTS•+ solution was diluted with methanol to obtain an absorbance of 0.70 ± 0.02 at 734 nm. For the assay, 40 µL of diluted CFS sample and 160 µL of the prepared ABTS•+ solution were combined in each well. The final CFS concentration ranged from 31.25% to 50% (v/v). After incubating the mixtures in the dark for 10 min at room temperature, the absorbance was measured at 734 nm using a microplate reader (Multiskan GO™, Thermo Scientific, USA). The percentage of ABTS radical scavenging activity was calculated using the following formula:

$$scavenging\;activity\left(\%\right)=\left[\right(Ac-As)/Ac]\times100,$$

where *Ac* is the absorbance of the ABTS•+ solution without CFS (negative control), and *As* is the absorbance of the reaction mixture containing the CFS sample. All measurements were performed in triplicate.

#### Ferric reducing antioxidant power (FRAP) assay

The ferric reducing antioxidant capacity of the LAB CFS samples was determined using the FRAP (Ferric Reducing Antioxidant Power) assay, following a previously reported method with minor modifications (Muhialdin et al. 2021). CFS samples were diluted to 25% (v/v) with distilled water, vortexed thoroughly, and centrifuged for 5 min at 4500 rpm to remove any residual cells or debris. The resulting supernatant was used for the assay.

The FRAP reagent was freshly prepared by mixing 300 mM acetate buffer (pH 3.6) (Merck, Germany), 10 mM 2,4,6 tripyridyl-s-triazine (TPTZ) (Sigma Aldrich, USA) solution in 40 mM HCl (Merck, Germany), and 20 mM FeCl₃·6 H₂O (Sigma Aldrich, St. Louis, MO, USA) at a ratio of 10:1:1 (v/v/v). In a 96-well microplate (Iwaki), 200 µL of the FRAP reagent was mixed with 20 µL of the prepared CFS samples. The reaction mixture were incubated in the dark at room temperature for 45 min. After incubation, absorbance was measured at 593 nm using a microplate reader (Multiskan GO™, Thermo Scientific, USA). A standard curve was constructed using ferric sulfate (FeSO₄·7 H₂O) (Merck, Germany) at concentrations ranging from 0 to 1000 µM. FRAP values of the samples were calculated from the standard curve expressed as µM Fe²⁺ equivalents. All measurements were performed in triplicate.

### α-Amylase inhibition assay of LAB cell-free supernatants (CFS)

The inhibitory ability of the LAB CFS against α-amylase was assessed using the method described by Huligere et al. (2023), with minor modifications. In this assay, porcine pancreatic α-amylase (Merck, Germany) was used as the enzyme source. A total of 100 µL of CFS was mixed with 100 µL of α-amylase solution (0.2 U/mL in PBS, pH 6.4), and the mixture was pre-incubated at 35 °C for 10 min. After pre-incubation, 200 µL of 1% (w/v) soluble starch solution prepared in 0.1 M PBS (pH 7.4) (Merck, Germany) was added to initiate the reaction. The reaction mixture was then incubated for another 10 min at 35 °C. To terminate the reaction, 200 µl of 3,5-dinitrosalicylic acid (DNS) reagent (Sigma MCLS, USA) was added to each tube, followed by incubation in a boiling water bath for 5 min. The tubes were subsequently cooled to room temperature, and 3 mL of distilled water was added to each tube. Absorbance was measured at 540 nm using a spectrophotometer (Cary 60 UV-Vis Spectrophotometer, Agilent, USA). The percentage of α-amylase inhibition was calculated using the following formula:

$$Inhibition\;rate\left(\%\right)=[1-(AbsB-AbsA\left)\right]\times100,$$

where *AbsA* is the absorbance of the control (without CFS), and *AbsB* is the absorbance of the test sample (with CFS present). All measurements were performed in triplicate.

### Total phenolic content (TPC) Assay of LAB cell-free supernatants (CFS)

The total phenolic content of the LAB CFS was determined using the Folin–Ciocalteu method, with gallic acid (GA) as the standard compound, following a previously reported procedure with minor modifications (Ayar-Sümer et al. 2024). To prepare a 1000 ppm gallic acid (Merck Millipore, Germany) stock solution, 1.3 mg of GA was dissolved in 1.3 mL of methanol. A series of GA standard solutions ranging from 0 to 240 ppm were prepared by appropriate dilution for construction of the calibration curve. For the assay, 20 µL of each GA standard or CFS sample (50% v/v in distilled water) was added to the wells of a 96-well microplate in triplicate. Then, 100 µL of Folin-Ciocalteu reagent (Merck Millipore, Germany) (previously diluted 1:10 with distilled water) was added to each well and mixed thoroughly. After a 5-minute incubation at room temperature, 80 µL of 20% (w/v) sodium carbonate (Na_2_CO_3_) (Merck, Germany) solution was added. The mixture was then incubated in the dark for 2 h at room temperature. After incubation, absorbance was measured at 750 nm using a microplate reader (Multiskan GO™, Thermo Scientific, USA). A calibration curve was generated by plotting the absorbance values against the corresponding concentrations of GA standards. The total phenolic content of the samples was calculated from the standard curve and expressed as gallic acid equivalents (GAEq) per mL of CFS. All measurements were conducted in triplicate.

### Total flavonoid content (TFC) of LAB cell-free supernatants (CFS)

The total flavonoid content of the LAB CFS was determined using a colorimetric assay based on a previously described method with modifications for microplate format (Islam et al. 2024). In a 96-well plate, 10 µL of 50% (v/v) CFS was mixed with 60 µL of methanol. Then, 10 µL of 1 M potassium acetate (CH_3_COOK) (Sigma-Aldrich, USA) and 10 µL of 10% (w/v) aluminium chloride (AlCl_3_) (Merck MCLS, Germany) were added. Finally, 120 µL of distilled water (Ika Pharmindo, Indonesia) was added to bring the total volume to 210 µL per well. The mixture was vortexed briefly and incubated at room temperature in the dark for 30 min. Absorbance was measured at 415 nm using a microplate reader (Multiskan Go™, Thermo Scientific, USA). Quercetin was used as a standard to construct the calibration curve, and total flavonoid content was expressed as milligrams of quercetin (Sigma Aldrich, USA) equivalent per mL of CFS (mg QE/mL CFS). All measurements were performed in triplicate.

### Metabolomic profiling using LC-HRMS analysis of LAB cell-free supernatants (CFS)

Metabolomic profiling of the LAB CFS was performed using ultra-high-performance liquid chromatography coupled with high-resolution mass spectrometry (UHPLC-HRMS). A freeze-dried aliquot of 2 mL of CFS was reconstituted in 2 mL of LC-MS grade methanol (Merck, Germany) and vortexed for 5 min to ensure complete mixing. The resulting solution was filtered through a 0.22 μm PTFE hydrophilic syringe filter and transferred into an HPLC vial for analysis.

Untargeted metabolomic analysis was conducted using a Thermo Scientific Dionex Ultimate 3000 RSLC Nano UHPLC system coupled with a Thermo Scientific Q Exactive mass spectrometer (Thermo Fisher Scientific, Massachusetts, USA). The chromatographic separation was performed using an Acclaim Vanquish C18 column (150 × 2.1 mm). The mobile phases consisted of solvent A (LC-MS grade water containing 0.1% formic acid) and solvent B (LC-MS grade acetonitrile containing 0.1% formic acid), with a flow rate of 0.3 mL/min and an injection volume of 5 µL. The gradient elution program was as follows: 0–16 min, 5% to 90% B; 16–30 min, isocratic at 90% B; 30–35 min, 90% to 5% B (re-equilibration).

Mass spectrometric detection was performed using electrospray ionization (ESI) in both positive and negative ion modes. The full MS resolution was set to 70,000, with a scan range of m/z 66.7–1000. Data-dependent MS/MS (dd-MS²) was acquired at a resolution of 17,500. Three technical replicate injections were analyzed per sample.

Raw data were processed using XCalibur software (Thermo Scientific). Metabolite features were selected based on the following criteria: mass error within ± 5 ppm and MS^2^ data acquired in DDA mode for the target ion. Metabolite annotation was performed using the MzCloud and ChemSpider databases.

### Data analysis

All experimental results, including antibacterial activity, probiotic properties, antioxidant activity, total phenolic content, total flavonoid content, and α-amylase inhibition, were analyzed statistically, each performed in triplicate, and the results are presented as mean ± standard deviation (SD). IC_50_ values for DPPH and ABTS radical scavenging assays were determined using GraphPad Prism v10.0.2 (GraphPad Software, USA). Nonlinear regression analysis was performed by plotting CFS concentration (% v/v) against the corresponding percentage inhibition to generate sigmoidal dose–response curves. Statistical comparisons were conducted using R version 4.1.3. One-way analysis of variance (ANOVA) was applied at a 5% significance level (*p* < 0.05), followed by Duncan’s multiple range test as a post-hoc analysis, implemented in RStudio (2024.04.02.764). Pearson’s correlation coefficients (r) calculated to examine linear associations among variables. Multivariate analyses, including principal component analysis (PCA) and hierarchical clustering analysis (HCA), were conducted using XLSTAT V2020.4.1.1020 (Addinsoft, France). PCA was applied as an exploratory multivariate approach to visualize overall patterns and relationships among biochemical and microbiological variables (LAB isolates). HCA results were visualized as dendrogram heatmaps to explore the relationships between LAB strains and their functional properties. Metabolomic data from UHPLC-HRMS were further processed using MetaboAnalyst 6.0 (https://www.metaboanalyst.ca/) (Pang et al. 2024). Analyses included peak intensity normalization, principle component analysis (PCA), and partial least squares discriminant analysis (PLS-DA) to visualize clustering patterns and identify discriminant metabolites.

## Result and discussion

### Screening and characterization of lactic acid bacteria

The isolation of LAB from seven types of SBH yielded 32 selected isolates, characterized as catalase-negative, Gram-positive, and capable of forming clear zones around colonies on MRSA medium supplemented with CaCO₃, indicating acid production. These isolates were subsequently screened for antibacterial activity against individual and mixed pathogenic bacterial strains. Five isolates, namely TB-3 from *Tetragonula biroi* honey, HI-1 and HI-5 from *Heterotrigona itama* honey, TS-4 from *Tetragonula sarawakensis* honey, and LT-3 from *Tetragonula laeviceps* honey, exhibited the highest inhibitory potential (Supplementary Table S1).

Molecular characterization based on 16 S rRNA gene sequence analysis showed that TB-3, HI-1, and TS-4 were closely related to *Lacticaseibacillus rhamnosus* strain NBRC 3425, with sequence similarities of 95.65%, 94.56%, and 94.64%, respectively, while HI-5 and LT-3 were similar to *Pediococcus acidilactici* strain DSM 20,284, with 93.48% and 91.53% identity, respectively. These results are consistent with previous findings in which *L. rhamnosus* GG, isolated from *H. itama* honey, demonstrated comparable antibacterial activity against various pathogens (Zulkhairi Amin et al. 2019), and *Lactiplantibacillus plantarum* SN13T, isolated from *T. binghami* honey, exhibited broad-spectrum inhibition against multiple pathogenic bacteria (Melia et al. 2022).

**Fig. 1:**
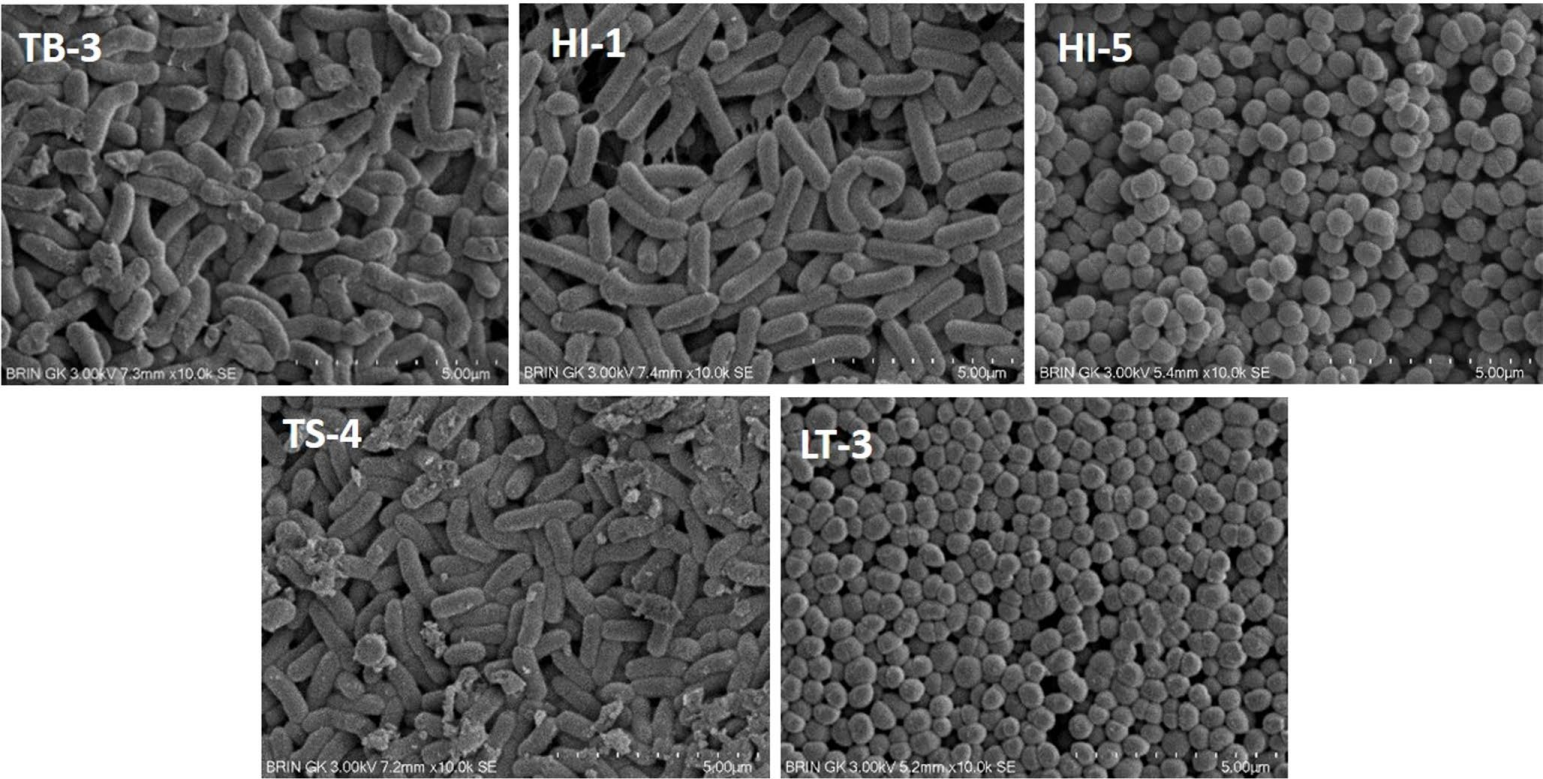
Scanning electron microscope images of lactic acid bacteria isolated from Indonesian stingless bee honey, captured at 10,000⋅ magnification. TB-3: *Lacticaseibacillus rhamnosus* TB-3, HI-1: *L. rhamnosus* HI-1, HI-5: *Pediococcus acidilactici* HI-5, TS-4: *L. rhamnosus* TS-4; LT-3: *P. acidilactici* LT-3

Morphological characterization using SEM supported the molecular identification, with isolates TB-3, HI-1, and TS-4 displaying rod-shaped morphology typical of *L. rhamnosus*, and isolates HI-5 and LT-3 exhibiting coccus-shaped cells characteristic of *P. acidilactici* (Fig. 1). Studies related to LAB isolated from commonly available Apis honey in the market have been reported; however, reports on LAB isolates from stingless bee honey are still limited. In previous research, the isolate *L. rhamnosus* was obtained from *Apis mellifera* honey (Hussain et al. 2025). *L. rhamnosus* was found in 30.77% of 88 honey samples from various geographical locations in Iran (Abadi et al. 2023). *P. acidilactici* from *A. mellifera* honey (Leska et al. 2022)d *acidilactici* HC isolated from Tualang honey in Malaysia (Bulgasem et al., 2016).

The 16 S rRNA sequences of these five isolates have been deposited in the GenBank database under the following accession numbers: PQ386496 (*P. acidilactici* LT-3), PQ386480 (*P. acidilactici* HI-5), PQ386429 (*L. rhamnosus* TB-3), PQ386482 (*L. rhamnosus* TS-4), and PQ386478 (*L. rhamnosus* HI-1). 16 S rRNA sequencing for genus-level identification has limitations in distinguishing closely related species or bacteria with low interspecies variation; moreover, 16 S-based identification largely depends on the quality of the reference database, so whole-genome sequencing (WGS) is still necessary to achieve accurate identification (de Souza et al. 2025). WGS and advanced analysis based on average nucleotide identity (ANI) are required as a powerful tool for high-resolution bacterial species identification (Rodriguez et al., 2024).

### Complete genome characterization

The genomes of five selected LAB isolates from stingless bee honey (SBH) were sequenced using the PromethION platform (Oxford Nanopore Technologies), and their key genomic features are summarized in Table 1. *L. rhamnosus* strains (TB-3, TS-4, and HI-1) exhibited genome sizes of approximately 2.98 Mb with a GC content of 46.8%, encoding 2,749; 2,748 and 2,754 coding sequences (CDSs), respectively. Although *L. rhamnosus* HI-1 and TS-4 have exactly the same genome size and GC content, they are independent individuals based on differences in the number of CDSs and ncRNA. Additionally, differences are also evident in further tests, such as differing ANI values compared to reference bacteria and the functional characteristics they possess. In contrast, *P. acidilactici* strains (HI-5 and LT-3) possessed smaller genomes (~ 1.96 Mb) with a GC content of 42.1% and 1,813–1,814 CDSs. All isolates harbored 56–62 tRNAs, one tmRNA, 15 rRNAs, 6–7 non-coding RNAs (ncRNAs), and one to two CRISPR elements. These genomic features are consistent with previous findings by Wang et al. (2025a, b), who reported that *L. rhamnosus* strain OF44 contained a genome size of 2.98 Mb, 2,791 CDSs, and a GC content of 47%, while *P. acidilactici* BCB1H exhibited a genome size of approximately 1.92 Mb, 1,895 genes, a GC content of 42.4%, and 192 annotated subsystems. The genome assemblies of all isolates from this study have been deposited in GenBank under the following accession numbers: CP184527 (*P. acidilactici* LT-3), CP184530 (*P. acidilactici* HI-5), CP184528 (*L. rhamnosus* TB-3), CP184531 (*L. rhamnosus* TS-4), and CP184529 (*L. rhamnosus* HI-1).

**Table 1:** Genome annotation of lactic acid bacteria isolated from stingless bee honey (*Tetragonula biroi*, *Tetragonula sarawakensis*, *Heterotrigona itama*, and *Lepidoptera terminata*)

Strain	TB-3	TS-4	HI-5	HI-1	LT-3
Genome size (bp)^1^	2,987,444	2,987,485	1,962,741	2,987,485	1,962,734
GC content^1^	46.8	46.8	42.1	46.8	42.1
Subsystems^1^	227	227	193	227	193
CDS^2^	2,749	2,748	1,814	2,754	1,813
tRNA^2^	62	62	56	62	56
tmRNA^2^	1	1	1	1	1
rRNA^2^	15	15	15	15	15
ncRNA^2^	7	7	6	7	6
CRISPRs sequence^2^	2	1	2	1	2

Annotation was performed using ^1^RAST server and ^2^Proksee software. GC, guanine-cytosine; CDS, coding sequence; tRNA, transfer RNA; tmRNA, transfer-messenger RNA; rRNA, ribosomal RNA; ncRNA, non-coding RNA; CRISPR, clustered regularly interspaced short palindromic repeats

**Fig. 2:**
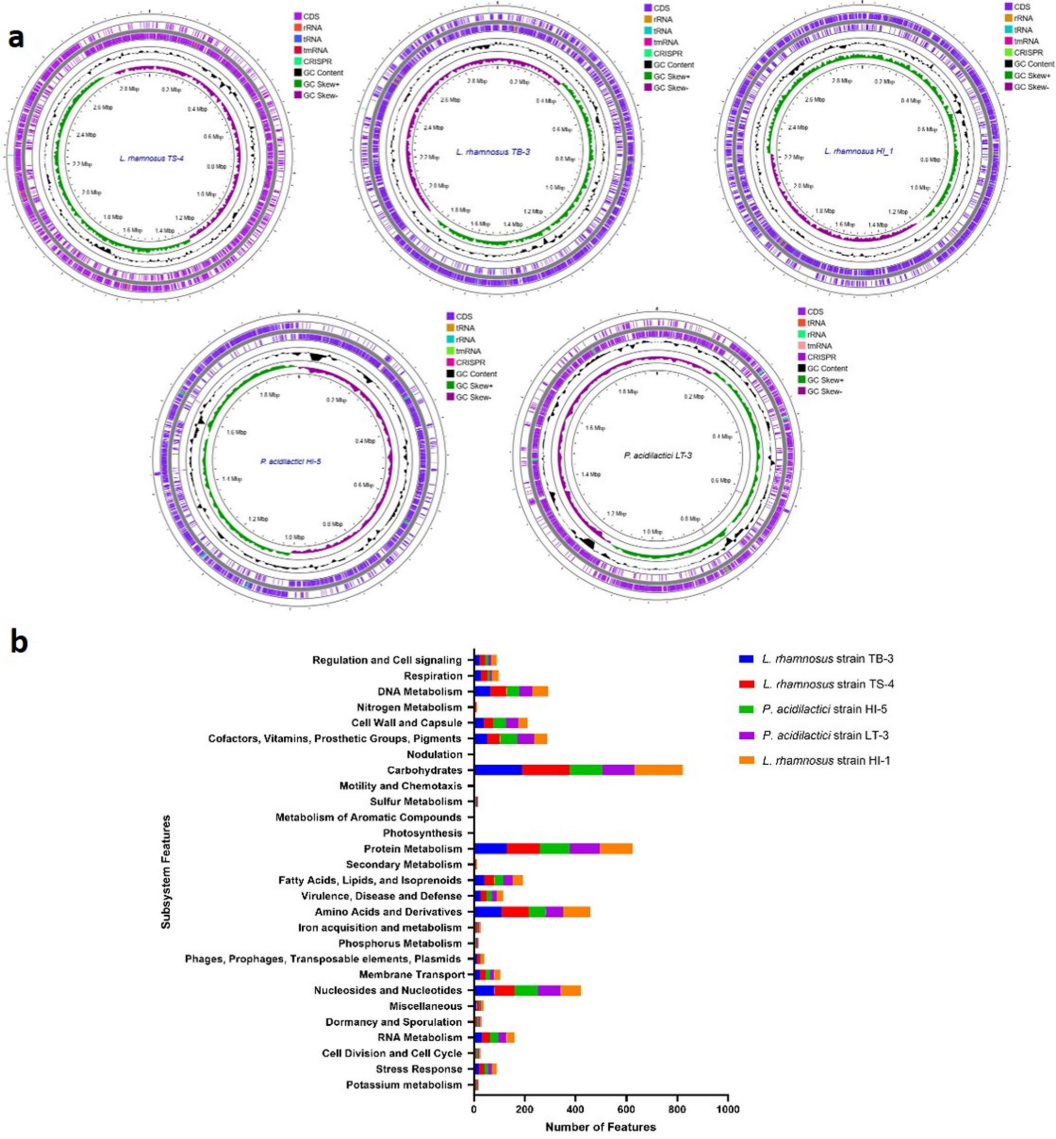
(**a**) Genome assembly and annotation of lactic acid bacteria isolated from Indonesian stingless bee honey. Circular genome maps were generated using Proksee (https://proksee.ca/). From the outermost to the innermost rings, the maps display coding sequences (CDSs), transfer RNA (tRNA), ribosomal RNA (rRNA), and transfer-messenger RNA (tmRNA). The central ring represents the complete genome, while the inner rings show GC content distribution on the positive and negative strands, respectively. (**b**) Functional classification of genes based on subsystem features, annotated using RAST server (https://rast.nmpdr.org/rast.cgi)

A graphical circular representation of the genomes and their functional subsystems is presented in Fig. 2a. Annotation using the RAST server and SEED Viewer revealed that the annotated genes were distributed across diverse biological pathways, including metabolic and regulatory subsystems (Fig. 2b). Subsystem analysis showed that the most enriched categories included carbohydrate metabolism, protein metabolism, amino acid and derivative metabolism, and nucleotide metabolism. In addition, genes associated with stress responses, virulence, defense mechanisms, and secondary metabolite biosynthesis were also annotated. The abundance of these genomic features highlights the functional diversity and potential of the LAB isolates for probiotic applications.

### Phylogenomic analysis

**Fig. 3:**
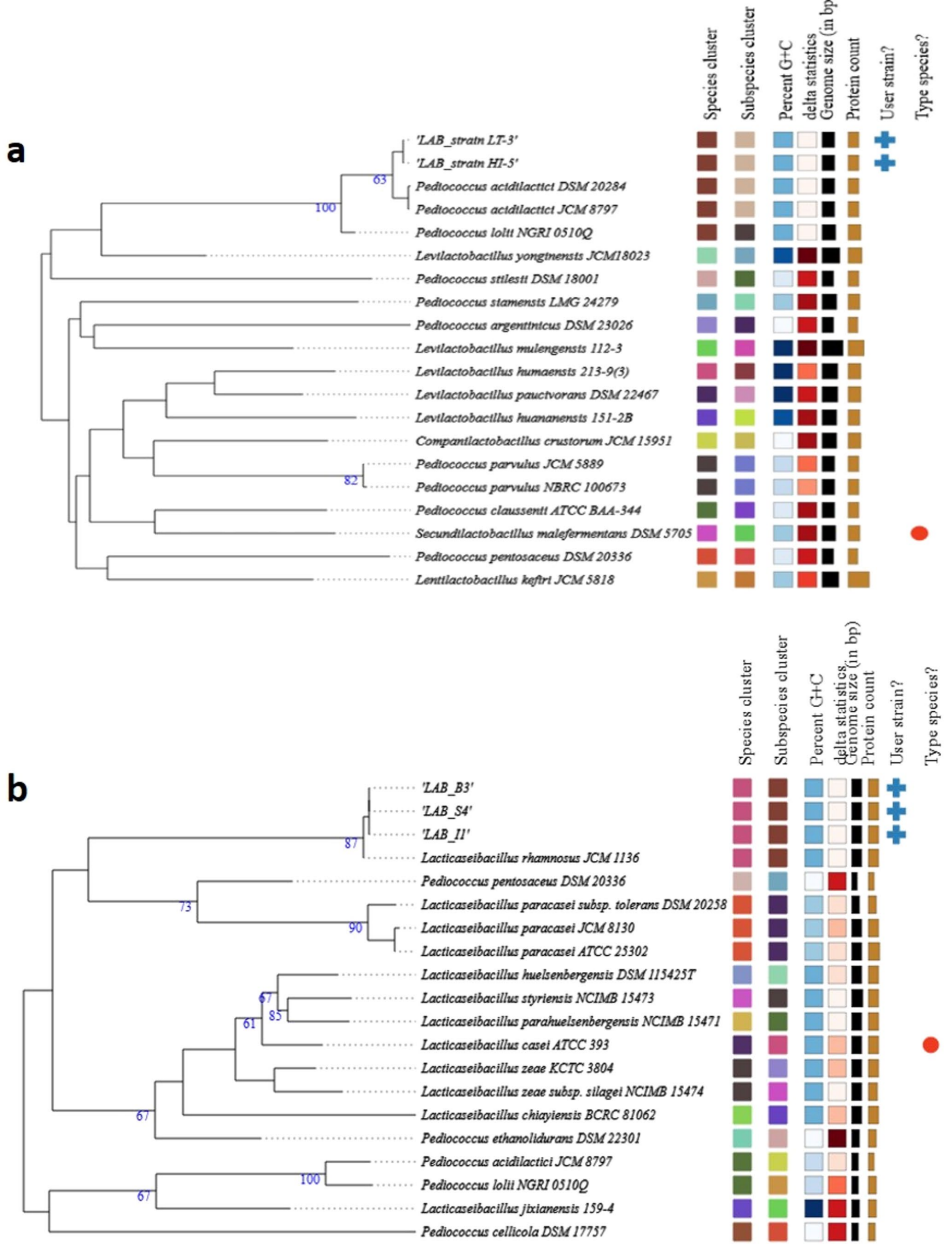
Phylogenomic tree of lactic acid bacteria isolates using the Genome BLAST Distance Phylogeny (GBDP) method based on whole-genome sequences, performed in FastME 2.1.6.1. Branch lengths are scaled using the GBDP distance formula d5. Bootstrap values above 60% (from 100 replicates) are shown; average branch support was 33.5%. The tree was rooted at the midpoint. (a) Clustering of isolates LT-3 and HI-5 within the *Pediococcus acidilactici* clade. (b) Clustering of isolates TB-3, TS-4, and HI-1 within the *Lacticaseibacillus rhamnosus* clade. Each tree includes reference type strains used for species-level comparison. The right panel summarizes additional genomic metrics for each strain, including species cluster, genome G + C content (%), digital DNA–DNA hybridization (dDDH, formula d4), average nucleotide identity (ANIb and OrthoANIu), and type strain status

Species- and subspecies-level identification was conducted using the TYGS genome analysis platform. Based on pairwise comparisons between the assembled genomes and type strain genomes, the taxonomic classification of the query strains is detailed in Tables S2 – S5. A total of 17 species emerged from the cluster analysis, with the LAB isolates assigned to distinct phylogenetic groups (Fig. 3). Isolates LT-3 and HI-5 clustered with *P. acidilactici* strains (Fig. 3a), while isolates TB-3, TS-4, and HI-1 grouped with *L. rhamnosus* strains (Fig. 3b).

For TB-3, TS-4, and HI-1, the highest digital DNA–DNA hybridization (dDDH, formula d4) values and the smallest GC content differences were observed in comparison with *L. rhamnosus* strain NCTC13764 (dDDH: 99.9%; GC difference: 0.08%). These isolates also showed OrthoANIu values of 99.89, 99.91, and 99.89, and ANIb values of 99.97, in comparison with *L. rhamnosus* DSM 20,021 (= JCM 1136). Based on genome-wide comparisons using the TYGS database and ANI values > 95%, these results confirm that TB-3, TS-4, and HI-1 belong to the *L. rhamnosus* species. The data of OrthoANIu and ANIb was displayed in Supplementary Table S6.

Similarly, isolates LT-3 and HI-5 exhibited dDDH values of 91.7% and a GC content difference of 0.07% with *P. acidilactici* DSM 20,284. OrthoANIu values were 99.05 and 99.07, while ANIb values were 98.89, also indicating high similarity with the reference *P. acidilactici* strain. These findings confirm that LT-3 and HI-5 are members of the *P. acidilactici* species, based on genome sequence analyses and ANI values exceeding the 95% threshold for species delineation.

### Genome mining analysis

Genome mining using AntiSMASH version 7 (Table 2) revealed that the L. rhamnosus group (TB-3, HI-1, and TS-4) harbored two biosynthetic gene clusters (BGCs): one encoding post-translationally modified peptides (RiPP-like), and the other encoding a type III polyketide synthase (T3PKS). Both clusters were relatively similar in length (41,173 nt for T3PKS and 19,553 nt for RiPP-like), but differed in sequence composition. According to MIBiG database comparisons, the RiPP-like cluster showed similarity to the coagulin gene cluster from *Bacillus coagulans* (ref. BGC0000617), while the T3PKS cluster resembled the viguiepinol biosynthetic gene cluster from *Streptomyces* sp. KO-3988 (ref. BGC0000286).

In contrast, *P. acidilactici* strains HI-5 and LT-3 contained distinct BGCs: an NRPS (non-ribosomal peptide synthetase) cluster in HI-5 and a T3PKS cluster in LT-3. Despite having similar lengths (53,770 nt), the two clusters showed distinct sequence compositions. Based on MIBiG comparison, both were homologous to the BGC encoding (2R,3 S,4 S)-5-fluoro-2,3,4-trihydroxypentanoic acid (5-FHPA) found in *Streptomyces* sp. MA37.

**Table 2:** Biosynthetic gene cluster (BGC) mining using antiSMASH version 7 and BAGEL4 of lactic acid bacteria genome isolated from Indonesian stingless bee honey

antiSMASH version 7				
Isolate	Cluster	Type	Location (start – end); total (nt)	MIBiG comparison (similarity score)
*L. rhamnosus* TB-3	Region 1	RiPP-like	893,042–912,594 (19,553)	Coagulin (0.34)
	Region 2	T3PKS	1,494,032–1,535,204 (41,173)	Viguiepinol (0.23)
*L. rhamnosus* HI-1	Region 1	RiPP-like	196,712–216,264 (19,553)	Coagulin (0.34)
	Region 2	T3PKS	797,702–838,874 (41,173)	Viguiepinol (0.23)
*L. rhamnosus* TS-4	Region 1	T3PKS	105,530–146,702 (41,173)	Viguiepinol (0.23)
	Region 2	RiPP-like	728,140–747,692 (19,553)	Coagulin (0.34)
*P. acidilactici* HI-5	Region 1	NRPS	20,445–74,214 (53,770)	(2R,3 S,4 S)-5-fluoro-2,3,4-trihydroxypentanoic acid (0.33)
*P. acidilactici* LT-3	Region 2	T3PKS	1,157,724–1,211,493 (53,770)	(2R,3 S,4 S)-5-fluoro-2,3,4-trihydroxypentanoic acid (0.33)
BAGEL4				
Isolate	Total bases in all DNA	Area of interest (AOI)	Start – end	Class
*L. rhamnosus* TB-3	2,987,444	1.0.AOI 01	882,992–908,842	51.2;Carnocin_CP52
*L. rhamnosus* HI-1	2,987,485	1.0.AOI 01	186,662–212,512	51.2;Carnocin_CP52
*L. rhamnosus* TS-4	2,987,485	1.0.AOI 01	731,892–757,742	51.2;Carnocin_CP52
*P. acidilactici* HI-5	1,962,734	Not found	-	-
*P. acidilactici* LT-3	1,962,741	Not found	-	-

T3PKS, type III polyketide synthase; NRPS, non-ribosomal peptide synthase; AOI, area of interest; RiPP, ribosomally produced and post-translationally modified peptides

Bacteriocin BGC prediction using BAGEL4 (Table 2) further indicated that all three *L. rhamnosus* strains (TB-3, HI-1, and TS-4) contained a region of interest (ROI) classified under the Carnocin class, although the genomic contexts varied among the isolates. Conversely, no bacteriocin BGCs were identified in the *P. acidilactici* strains HI-5 and LT-3, suggesting the absence of canonical bacteriocin biosynthesis clusters in their genomes.

Among the bacteriocins identified, Carnocin emerged as the most closely matched class. Particularly, the RiPP-like clusters detected in *L. rhamnosus* strains were similar to the coagulin gene cluster previously described in *Bacillus coagulans* I4. Coagulin is a protease-sensitive antimicrobial peptide categorized as a bacteriocin-like inhibitory substance (BLIS), exhibiting both bactericidal and bacteriolytic properties (Hyronimus et al., 1998). A study by Le Marrec et al. (2000) demonstrated that coagulin, a 44-residue peptide, shares 100% sequence identity with pediocin AcH and pediocin PA-1, both produced by *P. acidilactici* strains. The only difference lies in a single amino acid at the C-terminal end, making coagulin the first pediocin-like peptide reported from a non-lactic acid bacterium. These findings suggest that the antibacterial activity observed in *L. rhamnosus* isolates may be partially attributed to a coagulin-like compound encoded by their RiPP-like clusters.

In addition to RiPPs, the identified T3PKS cluster in *L. rhamnosus* also displayed homology to the viguiepinol gene cluster in *Streptomyces* sp. strain KO-3988 (Kawasaki et al. 2006). Viguiepinol [3-hydroxypimara-9(11),15-diene] is synthesized via the mevalonate pathway, a route commonly found in various prokaryotic species. Similarly, the NRPS cluster in *P. acidilactici* HI-5 showed similarity to the gene cluster encoding 5-FHPA, a novel fluorometabolite with potential antimicrobial activity produced by *Streptomyces* sp. MA37 (Ma et al. 2015). Furthermore, other members of the Carnocin class, such as carnocin UI49 from *Carnobacterium piscicola* UI49 and carnocin KZ213 from *C. piscicola* 213 (Khouiti and Simon 2004), have demonstrated bactericidal activity against *Listeria* spp., supporting their potential as biopreservatives (Stoffels et al., 1993). The diverse BGCs identified in these LAB isolates highlight their potential as probiotic candidates with biosynthetic capability to produce antimicrobial secondary metabolites.

### Probiotic properties

Following the initial screening that identified five LAB isolates with marked antibacterial activity, a comprehensive evaluation was carried out to further characterize their functional probiotic properties. This assessment included their tolerance to gastrointestinal stress, antioxidant potential, α-amylase inhibitory activity, and pathogen-specific antibacterial properties (Table 3).

Under simulated gastrointestinal conditions, all LAB isolates demonstrated notable tolerance to bile salts, gastric juice, and acidic pH, though with some variation in survival. All isolates maintained good viability (> 50%) after 1 and 3 h of bile salt exposure. Although *P. acidilactici* HI-5 exhibited the lowest mean viability at 3 h (48.06 ± 7.63%), this value was not significantly different from the other isolates. Particularly, *P. acidilactici* LT-3 showed the lowest tolerance at 1 h (82.16 ± 1.16%); however, all isolates still maintained survival rates exceeding 80%, indicating robust bile tolerance.

Similarly, exposure to simulated gastric juice resulted in minimal reduction in cell viability, with all isolates retaining survival rates above 60% after both 1 and 3 h of exposure. Among them, *L. rhamnosus* TS-4 exhibited the highest gastric tolerance at 3 h (71.53 ± 2.02%). Under acidic conditions (pH 2), all isolates maintained viability above 70% after 2 h of exposure, with *L. rhamnosus* TB-3 exhibiting the highest acid tolerance (75.68 ± 3.76%). These findings suggest that the tested LAB isolates exhibit promising probiotic potential based on their ability to withstand gastrointestinal stress conditions. This is predicted because the all bacteria possess CRISPR-Cas systems (Table 1), which indicates that probiotic bacteria have tolerance to environmental stress and enhances their survival and colonization, as well as their adaptation in the human digestive tract (Liu et al. 2023; Yang et al. 2020).

When compared with previous studies, the viability of these isolates under GI stress was slightly lower than that reported for *L. plantarum* TB1 isolated from *Tetrigona binghami* honey, which exhibited 94.44% bile salt tolerance and 82.75% acid tolerance (Melia et al. 2022). Likewise, *L. rhamnosus* GG isolated from *H. itama* honey maintained 97.46% and 106.76% cell viability under acidic and bile salt conditions, respectively (Zulkhairi Amin et al. 2019). In another study, four LAB strains isolated from *H. itama*, and *T. laeviceps* exhibited exceptionally high survivability, with acid tolerance at pH 2 (80.96 - 102.10%), bile salt tolerance after  1 h (86.86 - 10.50%), and gastric juice tolerance (80 - 97.00%) (Damayanti et al. 2025). *L. rhamnosus* (P0-2 L) isolated from *Apis mellifera* also has resistance to acidic conditions and exposure to gastric juice (Hussain 2025).

The resilience of LAB under gastrointestinal conditions has been attributed to the presence of specific stress-resistance genes. For example, genome analysis of *L. rhamnosus* strain OF44 identified nine genes associated with lysozyme resistance, eight with acid resistance, and 24 related to bile salt tolerance (Wang et al. 2025a, b). These genetic determinants are believed to confer adaptive advantages that enhance survival in hostile gut environments.

**Table 3:** Probiotic properties and cell-free supernatant activity on antioxidant, antibacterial and antidiabetic in vitro assay of lactic acid bacteria isolated from Indonesian stingless bee honey

Biological Activities	*L. rhamnosus* TB-3	*L. rhamnosus* HI-1	*P*. *acidilactici* HI-5	*L. rhamnosus* TS-4	*P*. *acidilactici* LT-3
Probiotic properties (% viability)					
Bile salt					
1-h	85.37 ± 0.83^a^	85.43 ± 0.43^a^	84.59 ± 0.52^a^	84.43 ± 1.09^a^	82.16 ± 1.16^b^
3-h	57.20 ± 4.51^a^	53.75 ± 3.80^a^	48.06 ± 7.63^a^	58.80 ± 2.49^a^	51.00 ± 4.60^a^
Gastric juice					
1-h	85.27 ± 1.10^a^	84.73 ± 0.93^a^	84.74 ± 3.64^a^	85.01 ± 1.64^a^	84.84 ± 1.98^a^
3-h	68.14 ± 3.69^a^	69.89 ± 0.90^a^	69.88 ± 5.53^a^	71.53 ± 2.02^a^	68.03 ± 5.98^a^
Low pH					
2-h	75.68 ± 3.76^a^	70.35 ± 5.08^a^	74.48 ± 4.76^a^	74.23 ± 4.54^a^	73.09 ± 4.12^a^
Antioxidant capacity					
DPPH assay (IC_50_) (% v/v)^b^	3.69 ± 0.06^a^	3.17 ± 0.09^b^	3.83 ± 0.11^a^	3.83 ± 0.53^a^	3.03 ± 0.10^b^
ABTS assay (IC_50_) (% v/v)^c^	4.79 ± 0.24^a^	3.85 ± 0.16^cd^	4.27 ± 0.23^b^	3.77 ± 0.07^d^	4.15 ± 0.16^bc^
FRAP assay (µg FeSO_4_/mL)^d^	373.19 ± 2.01^c^	381.69 ± 3.71^b^	362.32 ± 3.77^d^	426.55 ± 3.22^a^	343.27 ± 3.16^e^
TEAC assay (µg trolox/mL)^e^	49.94 ± 0.09^ab^	49.67 ± 0.15^bc^	50.19 ± 0.19^a^	48.68 ± 0.14^d^	49.57 ± 0.23^c^
Polyphenol (µg GAE/mL)^f^	238.22 ± 8.16^c^	271.51 ± 8.42^a^	213.55 ± 6.28^d^	277.87 ± 10.03^a^	254.09 ± 15.60^b^
Flavonoid (µg QE/mL)^g^	233.71 ± 5.99^a^	160.19 ± 4.61^b^	165.09 ± 5.38^b^	136.98 ± 2.43^c^	166.55 ± 2.43^b^
α-amylase inhibition (%)	24.80 ± 0.86^c^	26.17 ± 0.83^b^	56.78 ± 1.38^a^	23.15 ± 0.72^d^	57.44 ± 0.57^a^
Antibacterial activity (% inhibition)					
*Escherichia coli*	51.29 ± 1.38^b^	47.03 ± 1.32^b^	34.29 ± 4.34^c^	37.44 ± 2.00^c^	65.80 ± 8.38^a^
*Staphylococcus aureus*	46.51 ± 3.88^c^	55.98 ± 0.34^b^	44.95 ± 4.19^c^	54.67 ± 1.53^b^	62.67 ± 0.90^a^
*Pseudomonas aeruginosa*	74.40 ± 0.92^a^	55.98 ± 0.34^c^	44.95 ± 4.19^d^	54.67 ± 1.53^c^	62.67 ± 0.90^b^
Poly-bacteria	46.77 ± 7.94^c^	55.98 ± 0.34^ab^	44.95 ± 4.19^c^	54.67 ± 1.53^b^	62.67 ± 0.90^a^

Different letters within the same row indicate statistically significant differences (*p* < 0.05). DPPH: 1,1-diphenyl-2-picrylhydrazyl (DPPH); ABTS: 2,2’-azino-bis(3-ethylbenzothiazoline-6-sulfonic acid); FRAP, ferric reducing antioxidant power; TEAC, Trolox equivalent antioxidant capacity; GAE, gallic acid equivalent; QE, quercetin equivalent

For antioxidant capacity, the five LAB isolates exhibited distinct activities across multiple in vitro assays. The DPPH and ABTS assays, which are based on IC₅₀ values, measure radical scavenging activity, with lower values indicating greater efficacy. In contrast, the FRAP and TEAC assays assess reducing power and Trolox-equivalent antioxidant capacity, respectively, with higher values denoting stronger antioxidant potential. In the DPPH assay, *P. acidilactici* LT-3 and *L. rhamnosus* HI-1 exhibited the most potent radical scavenging activities, with IC₅₀ values of 3.03 ± 0.10% v/v and 3.17 ± 0.09% v/v, respectively, showing no significant difference between them. In the ABTS assay, *L. rhamnosus* TS-4 demonstrated the highest antioxidant activity (IC₅₀: 3.77 ± 0.07% v/v), significantly lower than most other strains, except *L. rhamnosus* HI-1. Consistently, *L. rhamnosus* TS-4 also showed the highest reducing power in the FRAP assay (426.55 ± 3.22 µg FeSO₄/mL). In contrast, the TEAC assay identified *P. acidilactici* HI-5 as having the greatest antioxidant capacity (50.19 ± 0.19 µg trolox/mL). These findings highlight the strain-dependent antioxidant potential of LAB isolates and support their applicability in oxidative stress-related functional food formulations.

Beyond their survival capacity, LAB also possess intrinsic antioxidant defense mechanisms, which may contribute to host health by mitigating oxidative stress. These include free radical scavenging activity and modulation of signaling pathways such as the nuclear factor erythroid 2-related factor 2 (Nrf2) and nuclear factor kappa B (NF-κB) (Gao et al. 2025). Whole-genome analyses of probiotic *Lactobacillus* species have identified genes encoding key redox-related enzymes, including NADH oxidase (NOX), NADH peroxidase, superoxide dismutase (SOD), Dps-like peroxide resistance proteins, and components of the thioredoxin reductase system (Feng and Wang 2020), highlighting their robust oxidative stress response systems.

The LAB isolates also demonstrated significant variability in total polyphenol content, total flavonoid content, and α-amylase inhibitory activity. Among the five isolates, *L. rhamnosus* TS-4 and HI-1 exhibited the highest concentrations of total polyphenols, with values of 277.87 ± 10.03 µg GAE/mL and 271.51 ± 8.42 µg GAE/mL, respectively. These two isolates did not differ significantly from each other (*p* > 0.05) but demonstrated markedly higher polyphenol levels compared to the other strains, particularly *P. acidilactici* HI-5, which recorded the lowest content at 213.55 ± 6.28 µg GAE/mL. For flavonoid content, *L. rhamnosus* TB-3 exhibited the highest concentration among all isolates, reaching 233.71 ± 5.99 µg QE/mL. Conversely, *L. rhamnosus* TS-4 displayed the lowest flavonoid level, at 136.98 ± 2.43 µg QE/mL with a statistically significant difference (*p* < 0.05). Meanwhile, for α-amylase inhibition, *P. acidilactici* LT-3 and HI-5 showed the highest activities at 57.44 ± 0.57% and 56.78 ± 1.38%, respectively, both significantly higher than those of the *L. rhamnosus* isolates, which ranged from 23.15% to 26.17%. This aligns with previous studies, where the CFS from the probiotic *P. acidilactici* FM-Pa-JXM94 demonstrated significant alpha amylase inhibition activity, ranging from 45.56% to 74.28% (Wang et al. 2025a, b). Similar to the results of this study, lower α-amylase activity was observed in the *L. rhamnosus* L1 - L4 isolates, which was only about 10–17.5% (Azadikhah et al. 2025). These results underscore the metabolic diversity among LAB isolates, suggesting that certain strains may provide enhanced functional benefits, particularly in polyphenol-related bioactivities and glycemic control through α-amylase inhibition.

The observed polyphenol and flavonoid contents are not solely attributable to residual substrates from the fermentation matrix but may also stem from the metabolic activities of LAB themselves. Several studies have reported the ability of LAB to synthesize and transform polyphenolic compounds. For instance, *Lactococcus lactis* has been shown to produce trans-resveratrol (Förster 2014), a bioactive polyphenol with numerous health benefits. Moreover, probiotic LAB can metabolize existing polyphenols during fermentation, thereby enhancing their bioavailability, generating novel bioactive intermediates, and increasing the total polyphenol content in fermented products (Feng and Wang 2020). LAB–flavonoid interactions are also diverse, including increased flavonoid concentrations, improved fermentation dynamics, and biotransformation of flavonoids into more bioactive or bioavailable forms (Park et al. 2021).

Futhermore, the five LAB isolates demonstrated varying levels of antibacterial activity against *Escherichia coli*, *Staphylococcus aureus*, *Pseudomonas aeruginosa*, and a mixed bacterial culture (poly-bacteria), as shown in Table 3. Among them, *P. acidilactici* LT-3 consistently exhibited the highest inhibitory activity across most pathogens, particularly against *E. coli* (65.80 ± 8.38%), *S. aureus* (62.67 ± 0.90%), and the poly-bacterial mixture (62.67 ± 0.90%), with statistically significant differences compared to the other isolates. Meanwhile, *L. rhamnosus* TB-3 showed the greatest inhibition against *P. aeruginosa* (74.40 ± 0.92%), significantly more effective than the remaining isolates. The other strains, such as *L. rhamnosus* HI-1 and TS-4, showed moderate activity across all tested pathogens, whereas *P. acidilactici* HI-5 had the lowest overall antibacterial effect. These results suggest strain-specific antibacterial capabilities among LAB isolates, with *P. acidilactici* LT-3 and *L. rhamnosus* TB-3 emerging as promising candidates for bio-preservative or antimicrobial applications targeting common foodborne and opportunistic pathogens.

The antibacterial and antioxidant activities of *L. rhamnosus* are predicted to be associated with the presence of the bactericidal agent Carnocin class (Khouiti and Simon 2004), antimicrobial (Hyronimus et al., 1998), and antioxidant Coagulin (Reddy et al. 2016), as well as viguiepinol, which belongs to the diterpenoid group and is potentially a contributing agent to the antimicrobial, antioxidant, and anti-inflammatory probiotic properties exhibited by the *Enterococcus casseliflavus* SHAMU-QH-02 strain (Li et al. 2025). This is confirmed by the presence of gene clusters encoding these compounds (Table 2). The antibacterial activity of *P. acidilactici* is predicted to be related to the presence of the antimicrobial agent (2R,3 S,4 S)-5-fluoro-2,3,4-trihydroxypentanoic acid (5-FHPA) (Ma et al. 2015); however, there is no literature proving the antioxidant effects of 5-FHPA. The antioxidant activity of *P. acidilactici* may originate from other metabolites.

**Fig. 4:**
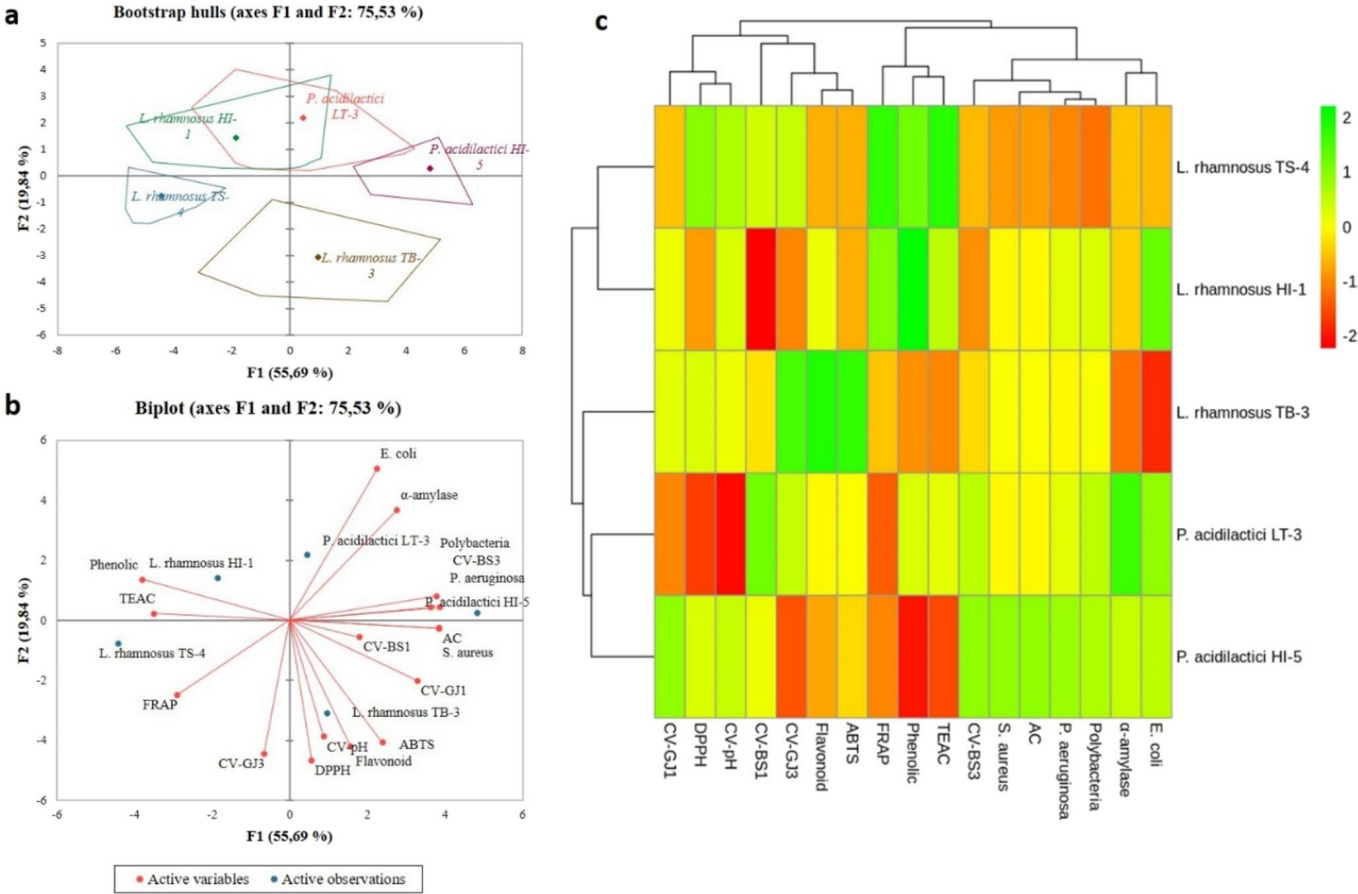
Multivariate analysis of functional characteristics of lactic acid bacteria (LAB) isolates derived from stingless bee honey. (**a**) PCA bootstrap hulls showing clustering of LAB isolates. (**b**) PCA biplot illustrating correlations between response variables and isolate distribution. (**c**) Heatmap with hierarchical clustering depicting the relative intensity of physicochemical and microbiological parameters across LAB isolates, with color gradients from red (lowest) to green (highest)

To illustrate the differences among LAB isolates based on physicochemical and microbiological parameters, PCA bootstrap hulls and biplots were generated (Figs. 4a–b). The first and second principal components accounted for 55.69% and 19.84% of the total variance, respectively, explaining a combined 75.53% of the variation. The bootstrap hull plot (Fig. 4a) showed distinct clustering of *P. acidilactici* LT-3 and *L. rhamnosus* HI-1, which were separated by a relatively small distance. The PCA biplot (Fig. 4b) further clarified the grouping patterns based on key functional attributes. Cluster 1 (*L. rhamnosus* HI-1) was associated with TEAC and total phenolic content, while Cluster 2 (*L. rhamnosus* TS-4) was mainly characterized by FRAP and was closely related to Cluster 1. Cluster 3 (*L. rhamnosus* TB-3) was defined by high DPPH and ABTS values, flavonoid content, and cell viability under gastric juice and acidic conditions. Cluster 4 (*P. acidilactici* HI-5) was distinguished by strong α-amylase inhibition, bile salt tolerance, and antibacterial activity, while Cluster 5 (*P. acidilactici* LT-3) showed no single dominant feature but exhibited proximity to Clusters 1 and 4.

This clustering pattern was further supported by the heatmap and hierarchical clustering analysis (Fig. 4c), which visually summarized the relationship between LAB isolates and their physicochemical and microbiological response variables. *L. rhamnosus* TS-4 and HI-1 were grouped together, both characterized by high FRAP, TEAC, and total phenolic content, indicating strong antioxidant potential. In contrast, *P. acidilactici* LT-3 and HI-5 formed a second cluster defined by strong α-amylase inhibitory activity and broad-spectrum antibacterial effects, yet showed lower DPPH and phenolic content. Meanwhile, *L. rhamnosus* TB-3 was placed in a distinct cluster, characterized by robust ABTS and elevated DPPH radical scavenging activities, high flavonoid content, and strong survival under gastric juice conditions. These visualizations underscore the strain-specific functional profiles of LAB isolates, highlighting their potential application in targeted probiotic formulations based on desired bioactivities.

To further explore these associations, Pearson correlation analysis was performed (Table S7). Antioxidant activity was primarily influenced by total phenolic content, as indicated by a strong positive correlation between phenolics and TEAC (*r* = 0.883). Flavonoid content was positively correlated with ABTS activity (*r* = 0.945), while α-amylase activity showed a positive correlation with bile salt tolerance (*r* = 0.836). Additional significant relationships were observed between FRAP and TEAC (*r* = 0.759), as well as between DPPH activity and viability under low pH conditions (*r* = 0.909).

### Metabolomic profiling of cell-free supernatant (CFS) compounds

The chromatographic profiles of the five LAB isolates are presented in Fig. S1. Distinct differences were observed between the metabolite profiles of *P. acidilactici* and *L. rhamnosus* isolates. Specifically, *P. acidilactici* HI-5 and LT-3 exhibited similar profiles, while *L. rhamnosus* HI-1 and TS-4 also clustered closely together, suggesting species-specific metabolic signatures. Interestingly, *L. rhamnosus* TB-3 displayed a slightly different chromatographic pattern, more closely resembling that of *P. acidilactici*, particularly at the initial retention time (highlighted with circles), indicating potential overlap in certain metabolites. The number of compounds in honey successfully analyzed using LC-HRMS was 262, and after a filtering process to eliminate duplicate compounds, 248 compounds remained (Supplementary Table S7). This data was then used for metabolomic analysis.

**Fig. 5:**
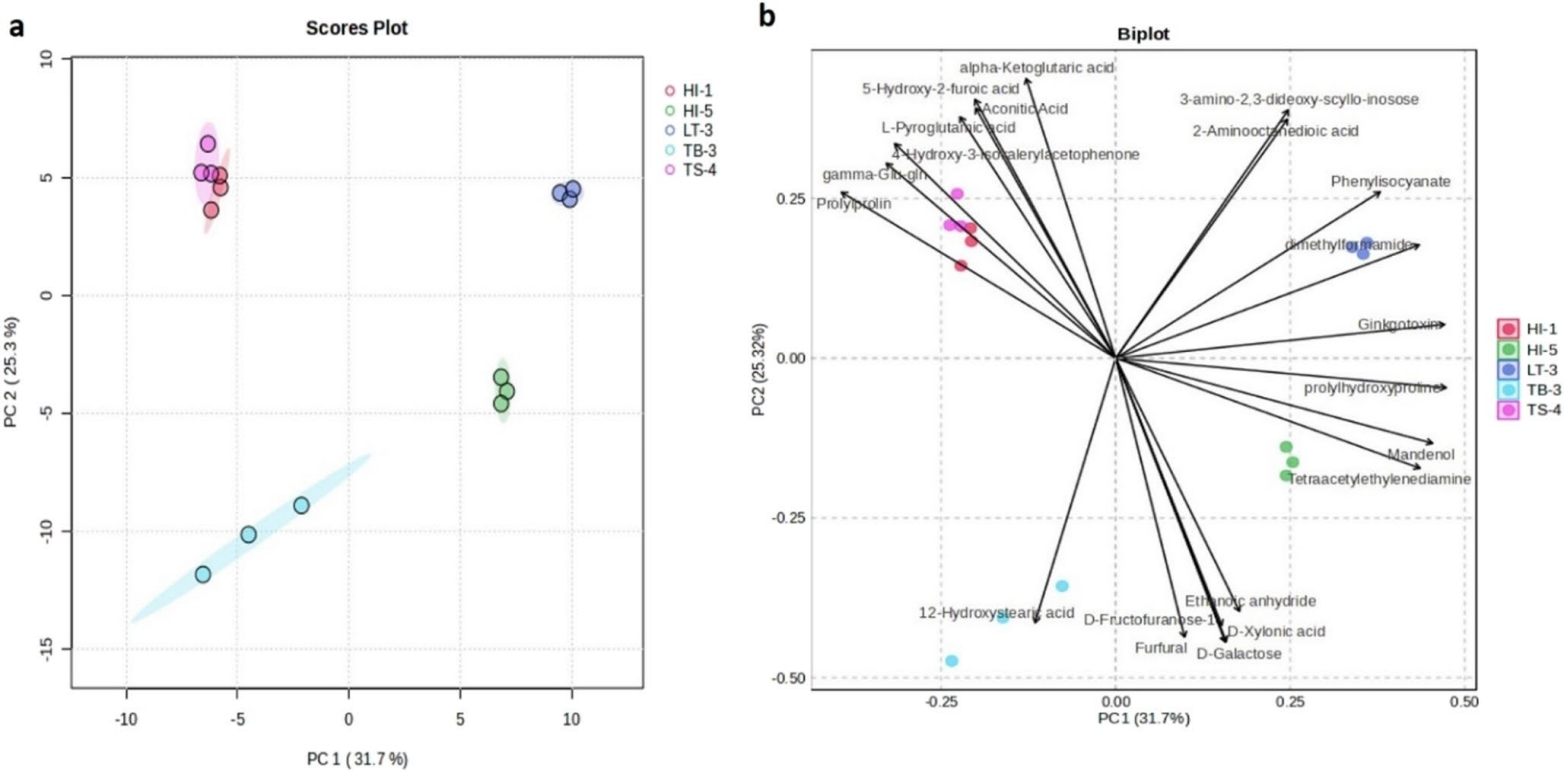
Metabolomic profiling of cell-free supernatant (CFS) compounds produced by lactic acid bacteria (LAB) isolated from Indonesian stingless bee honey, analyzed using LC-HRMS and multivariate statistics. (**a**) Principal Component Analysis (PCA) score plot showing the clustering pattern among five LAB isolates: *Lacticaseibacillus rhamnosus* TB-3, HI-1, and TS-4, and *Pediococcus acidilactici* HI-5 and LT-3. TB-3 appeared distinct, clustering closer to *P. acidilactici* isolates, suggesting a divergent metabolomic profile. (**b**) PCA biplot illustrating the top 20 discriminating metabolites contributing to strain differentiation. Specific metabolites, such as 12-hydroxystearic acid, were associated with TB-3, while other compounds like alpha-ketoglutaric acid, tryptophan, and phenylpyruvic acid were linked to functional activities of other LAB isolates. Data analysis was performed using MetaboAnalyst 6.0

These observations were supported by PCA (Fig. 5a), where *P. acidilactici* HI-5 and LT-3 formed one cluster, and *L. rhamnosus* HI-1 and TS-4 formed another, while *L. rhamnosus* TB-3 was distinctly separated, confirming its unique metabolic profile. Hierarchical clustering analysis (Fig. S2) further validated these groupings, showing consistent clustering of *L. rhamnosus* TS-4 with HI-1, and *P. acidilactici* LT-3 with HI-5, while positioning TB-3 closer to the *P. acidilactici* cluster, despite its taxonomic classification as *L. rhamnosus*.

Overall, the metabolite data revealed a strong agreement between chromatographic patterns, PCA, and clustering results. The biplot analysis (Fig. 5b) highlighted several discriminatory metabolites responsible for inter-strain variation. Among them, 12-hydroxystearic acid was identified as a unique feature of *L. rhamnosus* TB-3. These specific metabolites may contribute to the observed differences in bioactivity, particularly regarding strain-specific CFS functions, and underscore the importance of metabolic profiling in understanding functional characteristics of LAB isolates.

**Fig. 6:**
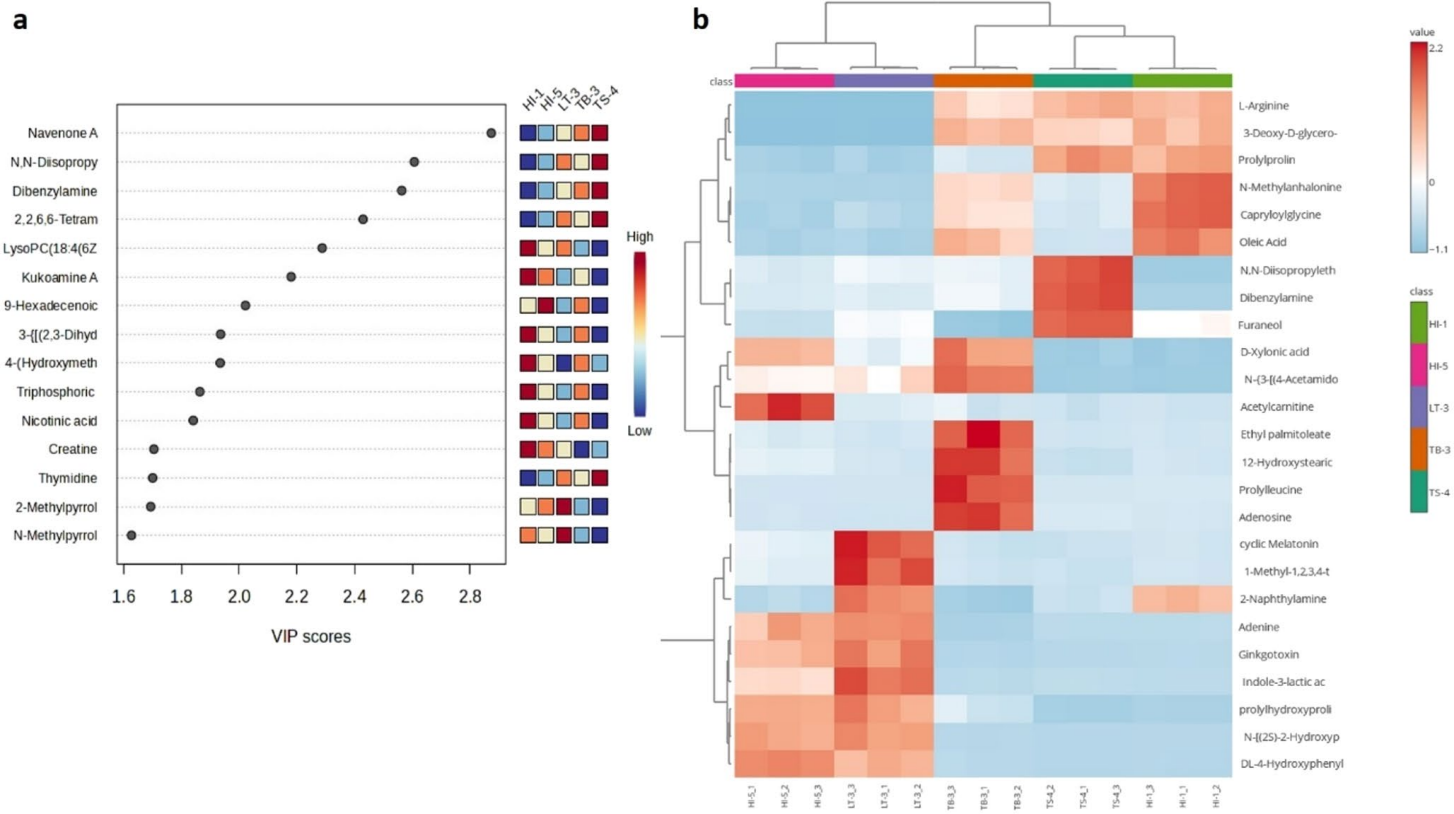
LC-HRMS metabolomic profiling of cell-free supernatant (CFS) lactic acid bacteria (LAB) isolated from honey produced by Indonesian stingless bees. **a**. Discriminated compound using Partial Least Squares Discriminant Analysis; **b**. Heatmap analysis between 25 top of compound features and LAB species. Data was generated using MetaboAnalyst 6.0. TB-3: *Lacticaseibacillus rhamnosus* TB-3, HI-1: *L. rhamnosus* HI-1, HI-5: *Pediococcus acidilactici* HI-5, TS-4: *L. rhamnosus* TS-4 and LT-3: *P. acidilactici* LT-3

To further identify the key metabolites distinguishing the five LAB isolates, PLS-DA was performed (Fig. 6a). The model revealed at least 15 compounds with Variable Importance in Projection (VIP) scores above 1.6, suggesting their significant contribution to strain differentiation. Among these, Navenone A, N, N-diisopropylethylamine (DIPEA), dibenzylamine, and 2,2,6,6-Tetramethyl-1-piperidinol had the highest VIP values (> 2.2). These metabolites showed low abundance in *L. rhamnosus* HI-1 but were enriched in *L. rhamnosus* TS-4.

The heatmap visualization (Fig. 6b) further supported the differentiation among LAB strains by illustrating distinct metabolite intensity patterns across isolates. *L. rhamnosus* TS-4 exhibited elevated levels of DIPEA, dibenzylamine, and furaneol, whereas *L. rhamnosus* HI-1 was enriched in N-methylanhalonine, capryloylglycine, and oleic acid. Meanwhile, *L. rhamnosus* TB-3 displayed a distinct profile characterized by high levels of D-xylonic acid, N-{3-[(4-acetamidobutyl)amino]propyl}acetamide, ethyl palmitoleate, 12-hydroxystearic, prolylleucine, and adenosine. Among the *Pediococcus* isolates, *P. acidilactici* HI-5 was notable for its abundance of cyclic melatonin and 1-methyl-1,2,3,4-tetrahydro-β-carboline-3-carboxylic acid, while *P. acidilactici* LT-3 was enriched in acetylcarnitine.

Across all isolates, 263 compounds were detected, with the top 10 based on area intensity listed in Table 4 (match > 72%). Citric acid was the most abundant metabolite, showing the highest peak area (12,743 × 10^6^), particularly in *L. rhamnosus* TS-4 and TB-3. Other dominant compounds included amino acids such as L-isoleucine, L-phenylalanine, valine, and L-arginine, along with 3-phenyllactic acid, a known lactic acid derivative.

Based on the compound area data (Table 4), *L. rhamnosus* TB-3, *P. acidilactici* HI-5, and LT-3 exhibited substantially higher levels of citric acid and L-isoleucine, ranging from 11,344 to 12,351 × 10⁶ and 9,627 to 11,078 × 10⁶, respectively, markedly greater than those found in *L. rhamnosus* HI-1 and TS-4. Citric acid is a well-documented antimicrobial agent (Scott et al. 2015; Ryan et al. 2019), while L-isoleucine is associated with anti-diarrheal properties (Alam et al. 2011) and glucose-lowering effects (Doi et al. 2005), which may relate to the α-amylase inhibitory activity observed in these isolates. These findings highlight that metabolite profiles and biological activity are driven more by strain-level differences than by species classification. Therefore, comprehensive characterization, including genomic, metabolic, and functional profiling, is critical to fully harness the probiotic potential of stingless bee-derived LAB.

**Table 4:** The top ten metabolites produced by lactic acid bacteria isolated from Indonesian stingless bee honey. The LC-HRMS instrument was used to analyze the metabolomic profiling

No.	Name	Formula	Calc. MW	RT [min]	Area (Max. 10^6^)	mzCloud Best Match	Area (Max. 10^6^)					Biological activities
							TB-3	HI-1	HI-5	TS-4	LT-3	
1	Citric acid	C_6_H_8_O_7_	192.02624	1.252	13,743	99.8	12,168	8,286	12,351	8,571	11,344	Antibacterial (Scott et al. 2015; Ryan et al. 2019)
2	L-Isoleucine	C_6_H_13_NO_2_	131.09451	1.423	12,027	99.9	11,078	7,608	10,168	7,556	9,627	Anti-Diarrhoea (Alam et al. 2011), blood glucose reduction (Doi et al. 2005)
3	L-Phenylalanine	C_9_H_11_NO_2_	165.07869	2.045	11,274	100.0	8,236	6,554	4,326	6,269	2,647	Antibacterial (Joondan et al. 2014), control blood sugar (Nuttall et al. 2006)
4	Valine	C_5_H_11_NO_2_	117.07901	1.128	4,225	96.9	3,909	3,213	3,974	3,200	3,952	Antimicrobial (Tsugami et al. 2023)
5	3-Phenyllactic acid	C_9_H_10_O_3_	166.06223	7.163	3,207	99.7	100	161	2,427	185	2,899	Antibacterial and antifungal (Xu et al. 2021)
6	L-Arginine	C_6_H_14_N_4_O_2_	174.11165	1.006	2,368	86.4	2,167	1,910	16	1,941	10	Antibacterial (Sepahi et al. 2017)
7	3-amino-4-(propylamino)cyclobut-3-ene-1,2-dione	C_7_H_10_N_2_O_2_	154.07416	2.062	2,232	62.5	1,979	1,401	1,914	1,050	1,843	-
8	Acetophenone	C_8_H_8_O	120.05758	1.345	1,898	72.4	890	881	997	796	1,823	Antibacterial (Sivakumar et al. 2008) antioxidant (Emami et al. 2018)
9	D-Galactoseis	C_6_H_12_O_6_	180.06269	1.054	1,746	97.4	1,387	137	1,009	167	513	Antibacterial and antioxidant (Zhang et al. 2022)
10	L-Pyroglutamic acid	C_5_H_7_NO_3_	129.04273	1.389	1,693	92.3	1,582	1,554	1,458	1,503	1,455	Antifungal (Ai et al. 2022)

TB-3, *Lacticaseibacillus rhamnosus* TB-3; HI-1, *L. rhamnosus* HI-1; HI-5, *Pediococcus acidilactici* HI-5; TS-4, *L. rhamnosus* TS-4; LT-3, *P. acidilactici* LT-3

This conclusion is further supported by integrated analysis across datasets. Genome-based prediction, phytochemical PCA (Figs. 4a–b), metabolomic PCA (Fig. 5a), and heatmap clustering (Fig. 6b) consistently revealed that *L. rhamnosus* HI-1 and TS-4 formed a cluster, particularly associated with antioxidant-related traits (TEAC, FRAP, TPC) and similar metabolomic signatures. The strong antioxidant activity in these isolates may be attributed to specific metabolites such as gamma-glutamylglutamine, furaneol, and oleic acid, previously reported for their antioxidant and antimicrobial properties (Dilika et al. 2000; Burgess et al. 2017; He et al. 2023).

Conversely, *L. rhamnosus* TB-3, *P. acidilactici* HI-5, and LT-3 showed enhanced biological activity (antioxidant, antibacterial, and acid/bile tolerance) (Fig. 4b), aligned with their distinct metabolite profiles identified in the biplot (Fig. 5b) and heatmap (Fig. 6b). Key metabolites such as 12-hydroxystearic acid and dimethylformamide have known cytoprotective and antimicrobial roles (Wang et al. 2001; Gowda et al. 2020). Additional compounds such as acetylcarnitine (HI-5), indol-3-lactic acid (LT-3), and ethyl palmitoleate (TB-3) have also been linked to antibacterial, anti-inflammatory, and gut health-promoting effects (Jirillo et al. 1991; Saeed et al. 2012; Meng et al. 2020; Wang et al. 2023). These patterns underscore the importance of strain-specific metabolomic fingerprints in shaping the functional potential of probiotic LAB candidates.

The unique antimicrobial and biochemical characteristics of lactic acid bacteria isolated from bees are also predicted to be influenced not only by the inherent factors of the originating species from the bee’s digestive tract, but also possibly related to LAB that may come from plant nectar, which serves as the food source for bees. Several types of LAB, such as *Lactobacillus*, *Pediococcus*, and *Fructobacillus*, have been found in fruits and flowers (Pimentel et al. 2021). Various types of lactic acid bacteria have also been isolated from different types of tropical fruits and flowers (Rodriguez et al., 2019). *L. plantarum* and *L. argentoratensis* has also been isolated from tropical fruits such as *Solanum nigrum*, *Couroupita guianenis*, and *Musa fruits* (Vasundaradevi et al. 2025). This opens up further research opportunities to confirm whether the lactic acid bacteria present in pure honey originate from the bee’s digestive tract or from the plant nectar consumed by the bees.

This study has successfully explored lactic acid bacteria species from the habitat of stingless bee honey, an area that has not been widely researched. In vitro tests of their potential as probiotic candidates, as well as the isolates’ ability to produce important metabolites with antibacterial, antioxidant, and alpha-amylase inhibition activities, are important as a foundation for further research. However, this study still has limitations regarding the application of probiotic candidates in food matrices and their ability to colonize the host’s digestive tract in vivo. Therefore, further research is needed to confirm their potential as probiotics in food that provide health benefits to the host.

## Conclusion

Five LAB isolated from Indonesian stingless bee honey (*Tetragonula biroi*, *T. sarawakensis*, *Heterotrigona itama*, and *Lepidoptera terminata*) showed significant antibacterial properties. *L. rhamnosus* (TB-3, HI-1, and TS-4) and *P. acidilactici* (HI-5 and LT-3), exhibited the strongest antibacterial activity and were selected for further characterization. These isolates demonstrated favorable probiotic attributes, including high survival rates under simulated gastrointestinal conditions (gastric juice, low pH, and bile salts). The CFS of these isolates showed strong antibacterial activity against common pathogens, significant α-amylase inhibitory activity, and robust antioxidant capacity as measured by DPPH, ABTS, and FRAP assays. These functional properties were supported by high polyphenol and flavonoid content. Untargeted metabolomic profiling via LC-HRMS revealed distinct bioactive metabolite signatures in the CFS of each isolate, underscoring the metabolic diversity of these strains. In addition, whole-genome sequencing of *L. rhamnosus* TB-3 identified genes associated with stress tolerance, antioxidant response, and biosynthesis of bioactive compounds. Altogether, the integration of genomic, metabolomic, and functional analyses provides strong evidence of the probiotic and postbiotic potential of these stingless bee-derived LAB strains. These findings support their promising application in the development of functional foods and nutraceutical formulations. While this research currently focuses on exploring potential probiotic candidates, it is essential to conduct further studies, including in vivo research, colonization tests in the digestive tract, and evaluations on food matrices.

## Supplementary Information

Below is the link to the electronic supplementary material.

Supplementary Material 1 (DOCX 18.7 KB)

Supplementary Material 2 (XLSX 28.7 KB)

Supplementary Material 3 (DOCX 18.3 KB)

Supplementary Material 4 (DOCX 23.5 KB)

Supplementary Material 5 (XLS 176 KB )

Supplementary Material 6 (JPG 159 KB)

Supplementary Material 7 (JPG 28.8 KB)

## Data Availability

All data generated or analyzed during this study are included in this published article and its supplementary information files. whole genome shotgun project was deposited in GenBank under the accession number CP184527 for *Pediococcus acidilactici* strain LT3, CP184530 for *P. acidilactici* strain HI5, CP184528 for *Lacticaseibacillus rhamnosus* strain TB3, CP184531 for *L. rhamnosus* strain TS4 and CP184529 for *L. rhamnosus* strain HI1.
