# Supplementary material for: Genomic, metabolomic, and functional properties of probiotic lactic acid bacteria isolated from Indonesian stingless bee honey
Source: Int Microbiol. 2026 Mar 13;29(4):509–31. doi: 10.1007/s10123-026-00794-4 (PMC13083383; doi:10.1007/s10123-026-00794-4)
Supplement: Supplementary file 1 — Supplementary Material 1 (DOCX 18.7 KB) [file 10123_2026_794_MOESM1_ESM.docx]

**Supplementary data Table S1**. Screening of antibacterial activity of lactic acid bacteria isolated from Indonesian stingless bee

| Stingless Bee Honey | Isolates code | % inhibitory activity at 30% of cell free supernatant concentration | | | |
| --- | --- | --- | --- | --- | --- |
|  |  | *E. coli* | *P. aeroginosa* | *S. aureus* | Poli-bacteria |
| *Heterotrigona itama* | HI-1 | 47.03 ± 1.31 | 58.49 ± 1.61 | 55.98 ± 0.34 | 58.81 ± 11.11 |
|  | HI-2 | 36.03 ± 1.37 | 31.48 ± 1.55 | 57.38 ± 1.51 | 40.66 ± 11.46 |
|  | HI-3 | 37.11 ± 2.38 | 28.84 ± 2.75 | 48.68 ± 6.03 | 39.73 ± 8.68 |
|  | HI-4 | 37.93 ± 2.52 | 31.62 ± 6.01 | 53.82 ± 1.29 | 38.43 ± 10.78 |
|  | HI-5 | 34.29 ± 4.34 | 27.13 ± 3.84 | 44.95 ± 4.19 | 47.10 ± 24.41 |
| *Tetragonula laeviceps* | TL-2 | 50.17 ± 18.69 | 36.29 ± 17.97 | 48.74 ± 0.63 | 37.82 ± 15.78 |
|  | TL-3 | 46.57 ± 5.12 | 34.97 ± 16.19 | 57.67 ± 4.13 | 44.03 ± 10.42 |
|  | TL-4 | 52.23 ± 16.98 | 37.10 ± 14.64 | 56.07 ± 4.52 | 38.11 ± 22.28 |
|  | TL-5 | 48.05 ± 9.87 | 35.45 ± 11.82 | 47.97 ± 5.34 | 43.51 ± 5.95 |
| *Trigona clypearis* | TC-1 | 58.73 ± 14.49 | 12.92 ± 4.64 | 61.70 ± 0.56 | 32.41 ± 32.84 |
|  | TC-2 | 34.74 ± 3.63 | 24.18 ± 3.59 | 62.08 ± 0.24 | 35.60 ± 18.57 |
|  | TC-3 | 35.67 ± 1.03 | -10.68 ± 7.13 | 64.97 ± 3.84 | 24.11 ± 32.29 |
|  | TC-4 | 35.07 ± 0.85 | -26.85 ± 11.19 | 60.87 ± 3.09 | 10.39 ± 44.65 |
|  | TC-5 | 34.12 ± 1.41 | -19.55 ± 7.18 | 58.54 ± 3.61 | 21.96 ± 32.97 |
| *Tetragonula sarawakensis* | TS-2 | 34.92 ± 1.09 | 13.58 ± 9.76 | 61.88 ± 0.13 | 28.94 ± 25.24 |
|  | TS-3 | 37.43 ± 2.39 | 24.62 ± 1.18 | 55.90 ± 2.64 | 34.81 ± 15.69 |
|  | TS-4 | 37.93 ± 2.52 | 22.96 ± 4.64 | 54.67 ± 1.53 | 42.91 ± 15.84 |
|  | TS-5 | 34.29 ± 4.34 | 24.35 ± 5.24 | 54.96 ± 0.85 | 38.42 ± 13.09 |
| *Lepidoptera terminata* | LT-1 | 49.61 ± 10.42 | 31.99 ± 13.04 | 58.34 ± 0.86 | 37.31 ± 21.66 |
|  | LT-2 | 52.61 ± 18.31 | 35.32 ± 14.33 | 61.34 ± 1.69 | 43.89 ± 15.96 |
|  | LT-3 | 65.80 ± 8.38 | 45.54 ± 4.03 | 62.67 ± 0.92 | 58.06 ± 8.91 |
|  | LT-4 | 39.40 ± 4.73 | 21.58 ± 1.18 | 57.49 ± 0.57 | 40.19 ± 14.73 |
|  | LT-5 | 38.68 ± 2.21 | 22.85 ± 1.33 | 61.35 ± 0.23 | 39.27 ± 16.16 |
| *Tetragonula dresceri* | TD-1 | 67.82 ± 0.46 | 42.55 ± 0.91 | 59.29 ± 0.21 | 48.00 ± 20.07 |
|  | TD-2 | 63.22 ± 2.51 | 24.87 ± 2.07 | 53.36 ± 1.52 | 42.20 ± 19.03 |
|  | TD-3 | 50.95 ± 28.45 | 22.55 ± 3.63 | 58.77 ± 0.21 | 36.63 ± 21.55 |
|  | TD-4 | 45.90 ± 22.31 | 41.08 ± 1.53 | 58.45 ± 1.02 | 41.05 ± 16.56 |
|  | TD-5 | 53.42 ± 9.34 | 23.44 ± 3.14 | 55.94 ± 3.83 | 40.79 ± 16.32 |
| *Tetragonula biroi* | TB-1 | 56.59 ± 2.55 | 56.48 ± 2.31 | 54.54 ± 5.38 | 51.67 ± 8.44 |
|  | TB-3 | 51.29 ± 1.38 | 46.77 ± 7.94 | 46.51 ± 3.87 | 54.74 ± 13.29 |
|  | TB-4 | 17.66 ± 2.74 | 25.89 ± 6.86 | 9.81 ± 5.48 | 26.74 ± 19.06 |
|  | TB-5 | 30.19 ± 1.06 | 37.43 ± 3.47 | 22.35 ± 17.03 | 34.09 ±10.25 |

All isolates have characteristic as negative catalase, and Gram-positive bacteria
