# Supplementary material for: Genomic, metabolomic, and functional properties of probiotic lactic acid bacteria isolated from Indonesian stingless bee honey
Source: Int Microbiol. 2026 Mar 13;29(4):509–31. doi: 10.1007/s10123-026-00794-4 (PMC13083383; doi:10.1007/s10123-026-00794-4)
Supplement: Supplementary file 3 — Supplementary Material 3 (DOCX 18.3 KB) [file 10123_2026_794_MOESM3_ESM.docx]

**Table 4.** Pairwise comparisons of *Lacticaseibacillus rhamnosus* TB-3, TS-4 and HI-1, *Pediococcus acidilactici* LT-3 and HI-5 and genome with the closest references genomes.

| **References (GenomeDB)** | **Size** | **Contigs** | **GC (%)** | **Ortho ANIu ^1^** | **Average Aligned Length (bp)** | **ANIb ^2^** | **Aligned**  **(%)** | **PCC ^3^** |
| --- | --- | --- | --- | --- | --- | --- | --- | --- |
| TB-3 | 2,987,444 | 1 | 46.76 |  |  |  |  |  |
| *Lacticaseibacillus rhamnosus* JCM 1136 (GCA_000615245) | 2,934,843 | 63 | 46.68 | 99.89 | 2,078,610 | 99.97 | 100.00 | 0.99959 |
| TS-4 | 2,987,485 | 1 | 46.76 |  |  |  |  |  |
| *Lacticaseibacillus rhamnosus* JCM 1136 (GCA_000615245) | 2,934,843 | 63 | 46.68 | 99.91 | 2,259,889 | 99.97 | 100.00 | 0.99959 |
| HI-1 | 2,987,485 | 1 | 46.76 |  |  |  |  |  |
| *Lacticaseibacillus rhamnosus* JCM 1136 (GCA_000615245) | 2,934,843 | 63 | 46.68 | 99.89 | 2,237,843 | 99.97 | 99.82 | 0.99959 |
| LT-3 | 1,962,741 | 1 | 42.06 |  |  |  |  |  |
| *Pediococcus acidilactici* DSM 20284 (GCA_000146325) | 1,942,827 | 4 | 42.13 | 99.05 | 1,243,759 | 98.89 | 90.00 | 0.99893 |
| HI-5 | 1,962,734 | 1 | 42.06 |  |  |  |  |  |
| *Pediococcus acidilactici* DSM 20284 (GCA_000146325) | 1,942,827 | 4 | 42.13 | 99.07 | 1,250,474 | 98.89 | 89.00 | 0.99893 |

1: Analysis of average nucleotide identity (ANI) was performed using the ANI calculator (<https://www.ezbiocloud.net/tools/ani>, accessed on 10 March 2025). 2: The ANI based on BLAST+, 3: correlation indexes of tetra nucleotide signatures (TETRA) using the JSpecies software tool version 4.2.3 (<https://jspecies.ribohost.com/jspeciesws/#analyse>, accessed on 10 March 2025).
