# Supplementary material for: Genomic, metabolomic, and functional properties of probiotic lactic acid bacteria isolated from Indonesian stingless bee honey
Source: Int Microbiol. 2026 Mar 13;29(4):509–31. doi: 10.1007/s10123-026-00794-4 (PMC13083383; doi:10.1007/s10123-026-00794-4)
Supplement: Supplementary file 4 — Supplementary Material 4 (DOCX 23.5 KB) [file 10123_2026_794_MOESM4_ESM.docx]

Supplementary data Table S7. Pearson’s correlation matrix between the measured parameters and variation of lactic acid bacteria (LAB) species from honey bee. Values in bold are different from 0 with a significance level alpha = 0.05. Abbreviations:
