# Supplementary figures and images for: Genomic, metabolomic, and functional properties of probiotic lactic acid bacteria isolated from Indonesian stingless bee honey
Source: Int Microbiol. 2026 Mar 13;29(4):509–31. doi: 10.1007/s10123-026-00794-4 (PMC13083383; doi:10.1007/s10123-026-00794-4)

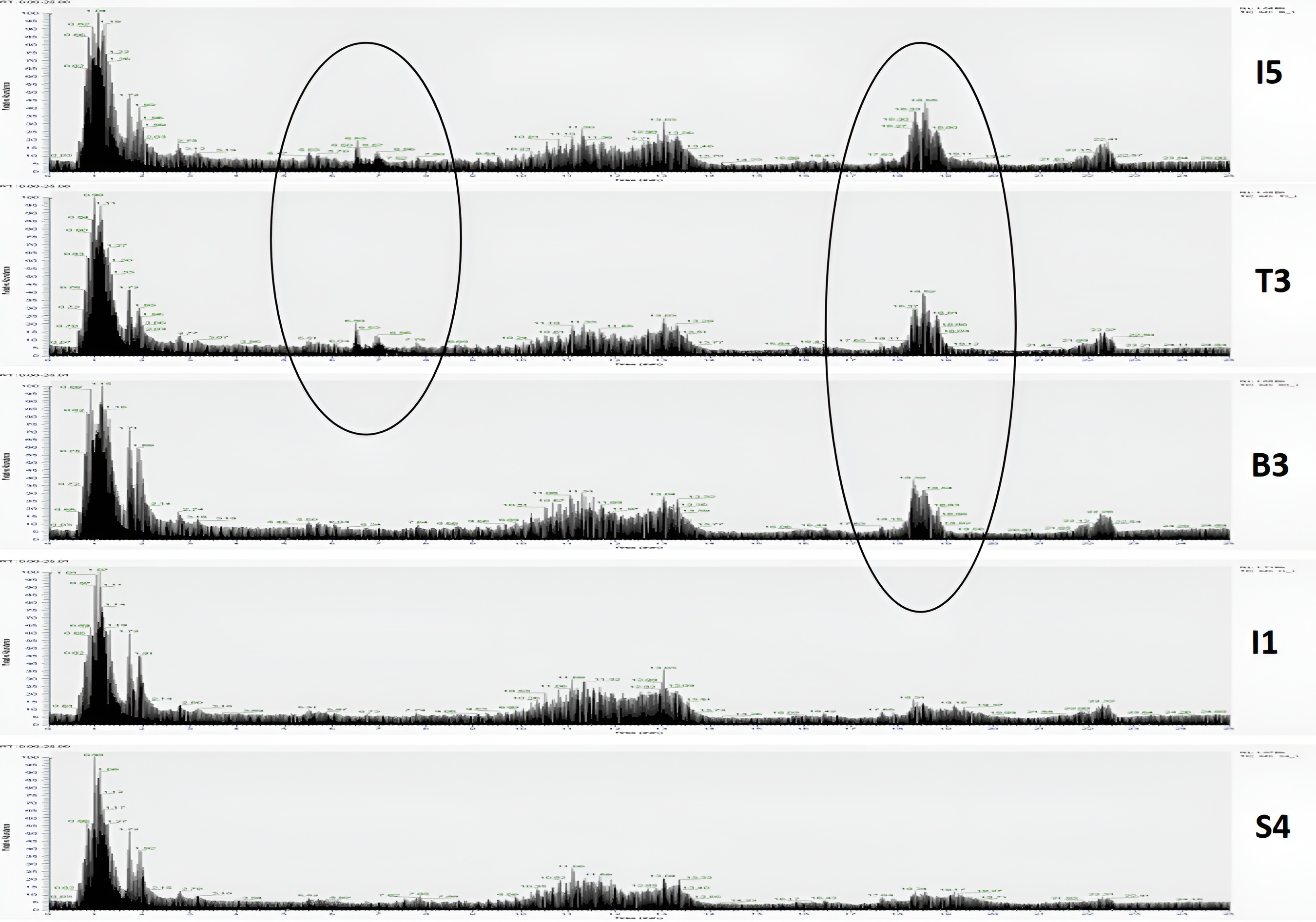

Supplement: Supplementary file 6 — Supplementary Material 6 (JPG 159 KB) [file 10123_2026_794_MOESM6_ESM.jpg]

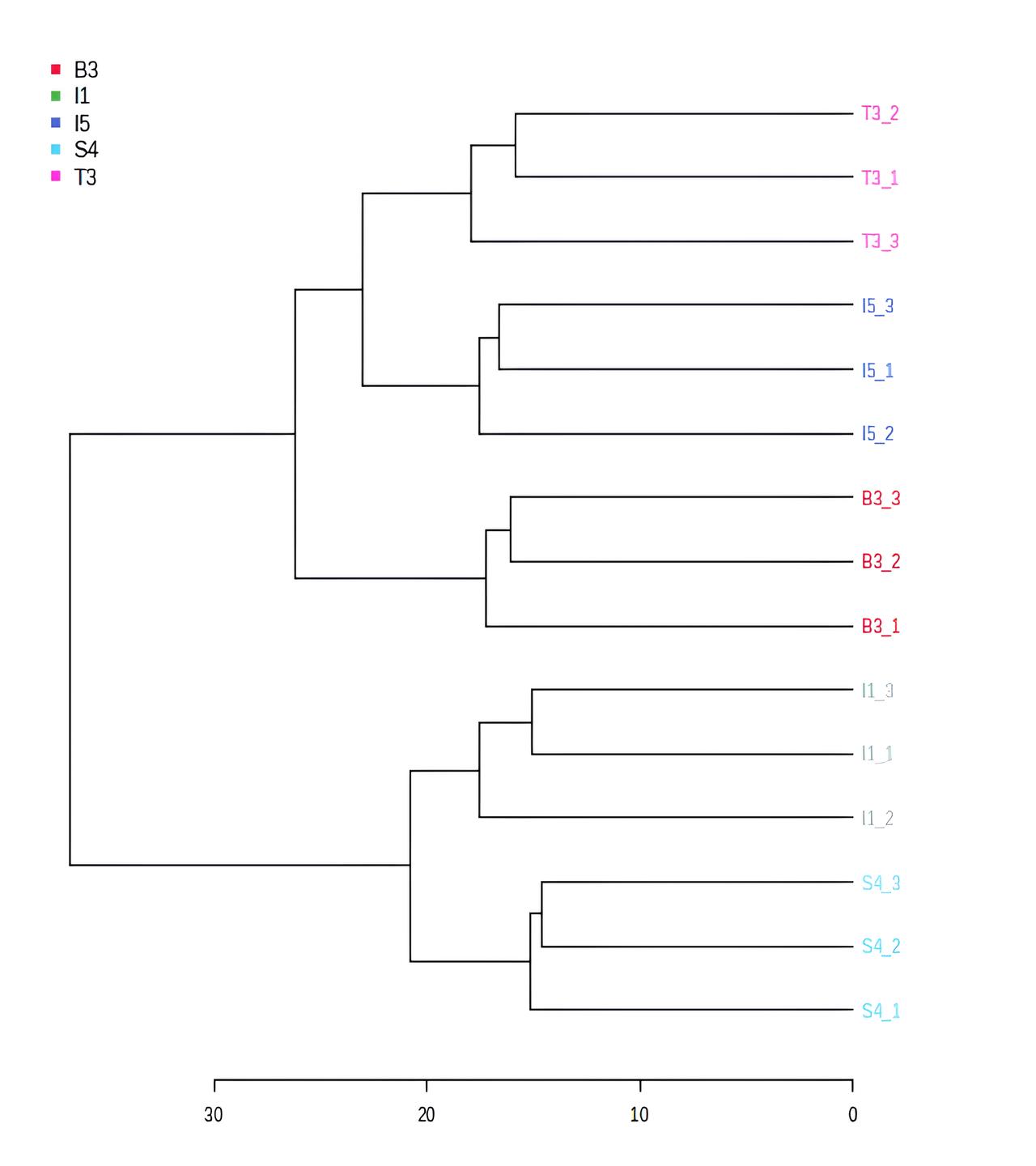

Supplement: Supplementary file 7 — Supplementary Material 7 (JPG 28.8 KB) [file 10123_2026_794_MOESM7_ESM.jpg]
